# Stochastic nucleation processes and substrate abundance explain time-dependent freezing in supercooled droplets

**DOI:** 10.1038/s41612-020-0106-4

**Published:** 2020-01-17

**Authors:** Daniel A. Knopf, Peter A. Alpert, Assaf Zipori, Naama Reicher, Yinon Rudich

**Affiliations:** 1 Institute for Terrestrial and Planetary Atmospheres, School of Marine and Atmospheric Sciences, Stony Brook University, Stony Brook, NY 11794-5000, USA; 2 Laboratory of Environmental Chemistry, Paul Scherrer Institute, 5232 Villigen, Switzerland; 3 Department of Earth and Planetary Sciences, Weizmann Institute of Science, Rehovot 76100, Israel

## Abstract

Atmospheric immersion freezing (IF), a heterogeneous ice nucleation process where an ice nucleating particle (INP) is immersed in supercooled water, is a dominant ice formation pathway impacting the hydrological cycle and climate. Implementation of IF derived from field and laboratory data in cloud and climate models is difficult due to the high variability in spatio-temporal scales, INP composition, and morphological complexity. We demonstrate that IF can be consistently described by a stochastic nucleation process accounting for uncertainties in the INP surface area. This approach accounts for time-dependent freezing, a wide range of surface areas and challenges phenomenological descriptions typically used to interpret IF. The results have an immediate impact on the current description, interpretation, and experiments of IF and its implementation in models. The findings are in accord with nucleation theory, and thus should hold for any supercooled liquid material that nucleates in contact with a substrate.

## INTRODUCTION

The formation of ice crystals in the atmosphere has important consequences for the radiative and precipitation properties of clouds, thus for the climate system and the hydrological cycle.^1,2^ The atmosphere represents a particularly challenging region to predict ice formation since ice nucleation occurs on nanometer- to micrometer-sized aerosol particles or in cloud droplets, commonly ~10 μm in diameter. As such, the available liquid volume or ice nucleating surface of an ice nucleating particle (INP)^3^ that initiates ice nucleation is miniscule. Predicting ice crystal formation on INPs remains a great challenge in climate models due to large uncertainties,^4,5^ despite the importance of ice formation in atmospheric processes and climate.

Different pathways exist for heterogeneous ice nucleation in the atmosphere.^3^ The dominant ice formation pathway in mixed-phase clouds, where ice crystals and supercooled droplets coexist, is immersion freezing (IF) where an insoluble INP is immersed in a supercooled water droplet.^6,7^ IF is also significant for cirrus cloud conditions.^6,8^ A conundrum in cloud microphysics studies is that the observed number of INPs does not match the observed number of ice crystals in clouds^6^ as is the case, e.g., for long-lived Arctic mixed-phase boundary layer clouds.^9^ Secondary ice formation processes may account for enhanced ice crystal numbers in some instances.^10^ However, laboratory and field observations are limited, and these secondary processes may only occur at specific temperature and droplet size ranges.^11–16^ Detailed cloud modeling studies indicate that ice activation should persist over long time to maintain the observed cloud structure and the amount of glaciation.^17–19^ In other words, continual ice nucleation may explain the observed discrepancy between INP number concentration and the actual ice crystal number concentrations with potential consequences for explaining observed cloud structure and precipitation amount. Online measurements of INP concentrations in a volume of air are commonly conducted by instantly exposing aerosol particles to supersaturated conditions with respect to water or ice at some temperature, *T*, for a short time (~10 s).^20,21^ This leads to cooling or humidification rates that are significantly faster than those experienced in the atmosphere, and lead to nucleation time scales much smaller than typical cloud formation time scales. Therefore, neglecting the time dependence of ice nucleation is not possible.

Laboratory ice nucleation data are often interpreted using a time-independent concept of ice nucleation active site (INAS) densities, *n*_s_, in units of cm^−2^. This mathematical normalization describes the number of observed nucleation events per unit INP surface area (ISA) at a given temperature, *n*_s_(*T*), and assumes that each nucleation event equates to one ice-active site.^7^ However, this framework neglects that nucleation is fundamentally a stochastic process based on fluxes of molecules to and from a critical cluster, and thus is inherently time dependent^22–24^ (see Supplementary Notes and Table 1).

The *n*_s_-based description employs an ad hoc parameter that can be used to compare studies, but in fact is not a physically-rooted parameter.^25^ As such, *n*_s_ is not a theoretically testable dependent variable. After decades of advancements in ice nucleation experiments and molecular dynamics simulations, a scientifically accepted framework that represents IF by INAS or describes which microscopic physicochemical parameters define an ice-active site has not yet emerged.^8^ Nanoscale characterization of particles is certainly difficult and has been the subject of entire studies.^26,27^ Despite great advances in spectro-microscopic analyses,^8,28–30^ observation of ice nucleation in situ on the nano-level (i.e., in the range of a few nanometers reflecting the critical cluster size) to unambiguously characterize ice-active sites has not been accomplished.^28,30,31^ Recent X-ray and electron spectro-microscopic observations of ice crystal formation on surfaces provide varying evidence. Ice crystals formed on preferable crystal faces of laboratory standard mineral particles^28^ and in surface defects on cleaved mineral sheets^30,31^ support the active site description. In the atmosphere, INPs and ice crystal residues are more physicochemically complex, and cover a range of particle types present in significant numbers in the ambient particle population.^32–34^ This supports the notion that the ice formation is also related to particle abundance or the total surface area. This exemplifies the challenge in linking surface characteristics of INPs to underlying ice nucleation physics. Clearly, different surfaces can interact with the surrounding water molecules differently.^6–8,35,36^ However, application of an arbitrary variation of these properties in the form of different *n*_s_ values to describe the temperature or time dependence of IF of same INP type is not theoretically established, thus eliminating to have a predictive framework for atmospheric ice nucleation.

Nucleation theory invokes a homogeneous and heterogeneous ice nucleation rate coefficient, *J*_hom_ in units of cm^−3^s^−1^, and *J*_het_ in units of cm^−2^s^−1^, respectively, to account for the dependence of nucleation probability on liquid volume or ISA and time.^22,23^ As a direct result one obtains when increasing ISA, the probability to nucleate ice increases exponentially.^22,23^ Classical nucleation theory (CNT) extends nucleation theory by assuming a macroscopic density and surface tension of water molecular clusters with their energy following a Boltzmann distribution.^23^ In the case of pure water droplets, *J*_hom_ and *J*_het_ depend on *T*, while in aqueous solutions, they additionally depend on solute concentration or water activity.^25,37–39^ In other words, for a given temperature, water activity, and INP type, one *J*_het_ value exists.

**Fig. 1:**
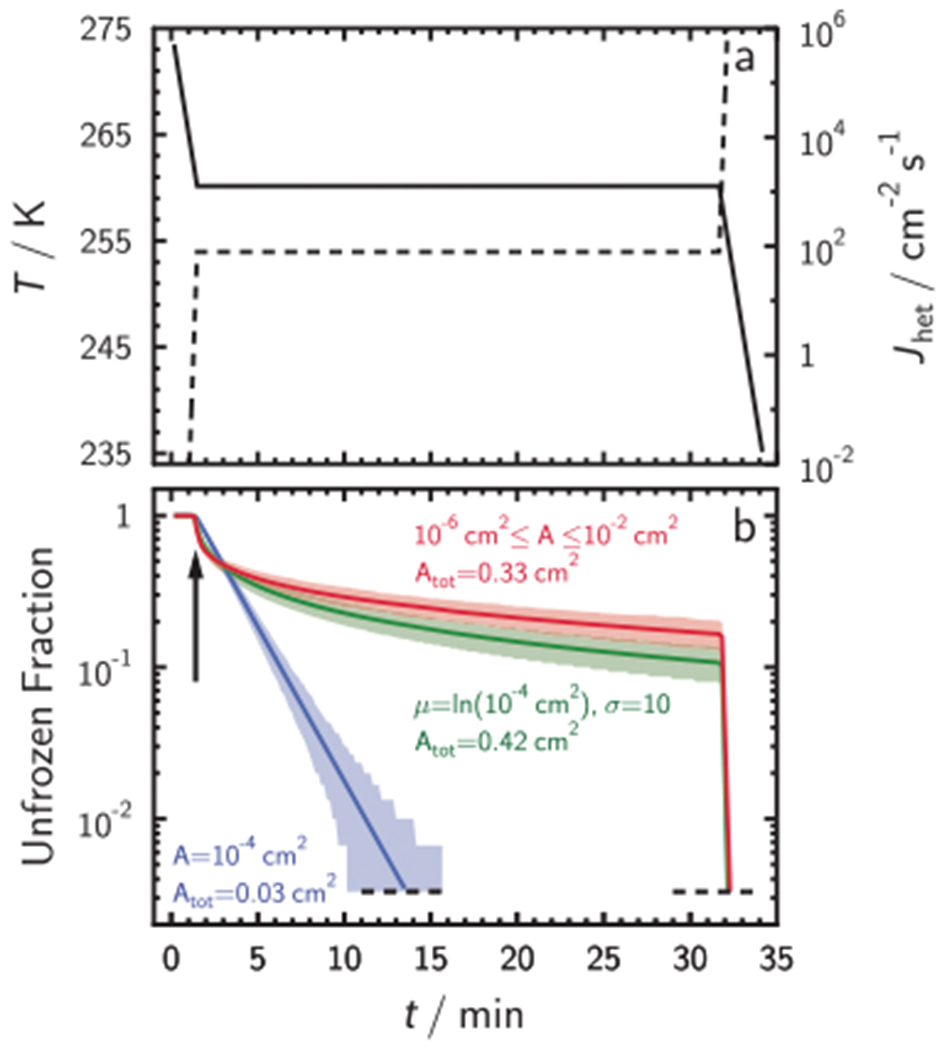
Isothermal immersion freezing experiment. **a** A typical experimental temperature, *T*, profile over time, *t*, (solid line) where the droplets are held constant after an initial cooling ramp. At about *t* = 31 min, *T* is further decreased to induce freezing in all droplets. Dashed line shows an example of the corresponding heterogeneous ice nucleation rate coefficient, *J*_het_. **b** Exemplary profile of the unfrozen droplet fraction as a function of t derived from simulating freezing using 300 droplets. The blue solid line represents simulated unfrozen droplet fraction assuming identical ice nucleating particle surface area, ISA, of *A* = 10^−4^ cm^2^ in each droplet. The green line represents the case of a lognormally distributed ISA around *A*, with *σ* = 10. The red line is a case where ISA is uniformly distributed by ±2 orders of magnitude in ISA around *A*. All curves apply the same *J*_het_ depicted in **a**. Shaded areas represent 5th and 95th percentiles.^22,25^ Total surface area, *A*_tot_, is indicated. The arrow indicates the *t* from which on the *T* is constant. Horizontal dashed lines indicate the limit of detection equal to 1/300 droplets.

There is a clear experimental evidence for a time-dependent behavior of IF,^7,24,25,39^ shown conceptually in Fig. 1a illustrating cooling of a sample of water droplets containing the same INP type to a target temperature that is kept constant for 30 min. This experiment is termed isothermal immersion freezing (ISO). With time, the droplets continuously freeze as indicated by the decrease of the fraction of unfrozen droplets, UnF (Fig. 1b). Since ns is time-independent, inherently it cannot explain continuous freezing under isothermal conditions. Doing so would lead to large predictive uncertainties as shown in Supplementary Fig. S1, which would not arise when using *J*_het_. It is, hence, suggested that application of *J*_het_ that captures the time dependence of nucleation would be a superior approach to describe ISO experiments. From observations of nucleation, one derives^22,23,25^

$$\text{UnF}(T)=\frac{N_{\text{ufz}}(T)}{N_{\text{tot}}}=e^{-J_{\text{het}}(T)At},$$

where *N*_ufz_ is the number of unfrozen droplets, *N*_tot_ is the total droplet number examined, *A* is the ISA in a droplet, and *t* is the time the droplets stay liquid. For the same ISA in each droplet and a fixed *J*_het_, Eq. (1) states that ln(*N*_ufz_/*N*_tot_) versus time should follow a straight line for ISO experiments as illustrated in Fig. 1b, blue line. According to CNT, isothermal homogeneous freezing of water droplets of equal volume will also follow a straight line as verified by our experiments (see Supplementary Methods and Supplementary Figs S2 and S3). However, UnF in some previous ISO experiments^25^ have been observed to be curved as indicated by the green and red curves in Fig. 1b. This was previously interpreted as a consequence of different active sites in INPs immersed in and among the investigated droplets that contain the same INP material.^7,8^ However, UnF results deviating from Eq. (1) could be a consequence of different ISA per droplet and thus questioning the validity of using Eq. (1) for data interpretation.^25^ Supplementary Fig. S4 presents the case of constant cooling rate IF (CCR) experiments for same ISA distributions shown in Fig. 1 including the display of frozen fraction (FF), *J*_het_, and *n*_s_.

As demonstrated by many laboratory measurements,^7,8,35,39^ when ISA increases, the probability to nucleate ice also increases. Contrary to assumptions of previous studies, we put forth that any experiment examining freezing droplets with immersed INPs cannot prepare them with identical ISA. In other words, the distribution of ISA per droplet will have an uncertainty or variability and therefore, the ice nucleation probability will also vary between the droplets. We only assume that the material- specific surface dominating features that make one material a better or worse INP compared to others, increase by the same amount as ISA increases and nucleation occurs randomly. We do not consider varying ice-active sites on the ISA per droplet. This alternative approach to interpret data from ISO and CCR experiments avoids empirical distributions of INAS (see Supplementary Notes). Below we present an interpretation of IF data using the underlying axioms of nucleation theory and physical observables, thus allowing to test and quantify this approach. In other words, we will separately assess the role of the stochastic nature of nucleation in IF and how variance of ISA per droplet impacts our understanding of IF.

We unambiguously demonstrate that IF follows the stochastic nature expected from nucleation theory and discuss the importance of accurate knowledge of the ice nucleating surface features to infer the underlying ice nucleation processes. Previously, analysis of IF studies was performed either from individual CCR or ISO experiments.^25^ In this study, we have used a microfluidic apparatus to control droplet volumes of a large number of droplets. This allows to represent CCR and ISO-derived IF data in one global simulation, which yields a tighter constraint on ice nucleation kinetics compared to previous approaches. It will be demonstrated that accounting for variance in the ISA can explain observed trends in IF data. Our findings impact the interpretation and parameterization of IF, question the prevailing concept of ice-active sites, affect experimental procedures and design for field and laboratory measurements, have ramifications for the development of a standard INP and finally, allow us to estimate the smallest achievable uncertainty to predict IF relevant for cloud and climate models. Lastly, this approach should hold for any nucleating supercooled liquid in contact with a solid that initiates nucleation and, as such, should be applicable to a wide range of disciplines.

## RESULTS

### Variability of ISA

As previously mentioned, Fig. 1b shows UnF for three example ISO experiments using identical ISA yielding a log-linear relationship and using lognormally and uniformly distributed ISA that both deviate from a log-linear relationship. On a nanoscopic level (crack, pores, and cavities on the nanoscale), differences in ISA between different particles in different liquid samples exist. Therefore, it is not possible to assume that the ISA among droplets is identical even when droplets are carefully prepared in the laboratory.^25^ The assumption of identical ISA in water droplets was examined by evaporation of water from 0.2 μL droplets containing 0.001 wt% NX illite.

Although the lognormally distributed ISA per droplet has an order of magnitude greater total ISA compared to the case of using identical ISA, the UnF remains much larger (Fig. 1b). Larger ISA should result in a larger freezing probability and a lesser UnF (i.e., more droplets froze), but according to Fig. 1b this is not necessarily the case. The unequal distribution of ISA among the droplets can cause some to contain very large ISA that freeze quickly (after the first 3 min in Fig. 1b) whereas droplets with small ISA stay liquid for longer time. In other words, the loss of surface area is disproportionally large when the first freezing events occur compared to later freezing events. Allowing for ISA variability and uncertainty in droplet freezing experiments warrants a reevaluation of nucleation theory and adoption of INAS densities to explain the trends in UnF observations. We show below for ISO and CCR experiments that observed UnF and FF, respectively, are consistent with ice nucleation kinetics when the ISA variability is accounted for. Furthermore, the variance in our freezing data can be solely explained by the stochastic uncertainty of freezing, thus excluding unexplained uncertainty attributable to unknown or arbitrarily defined INAS.^25^

### ISO and CCR freezing experiments and simulations

**Fig. 2:**
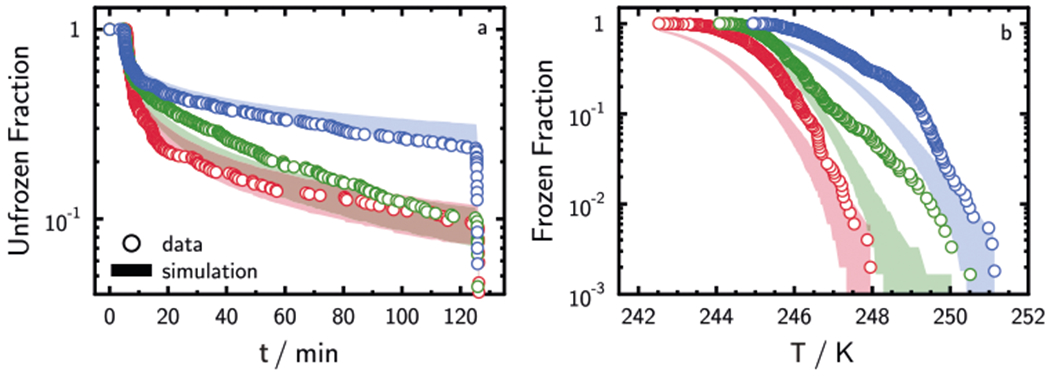
Isothermal and constant cooling rate immersion freezing experiments. Experimentally derived (symbols) and simulated (shading) unfrozen fractions of isothermal freezing experiments (**a**) as a function of time, *t*, and frozen fractions of constant cooling rate freezing experiments (**b**) as a function of temperature, *T*, outlined in Supplementary Table S1. Immersed surface area per droplet is unknown and lognormally distributed in model (see Supplementary Table S2). Blue color represents ISO1 and CCR1, green color represents ISO2 and CCR2, and red color represents ISO3 and CCR3. Shadings represent the modeled 5th and 95th percentiles.

Freezing of water droplets containing illite was performed for three sets of ISO experiments and three sets of CCR experiments (see Supplementary Table S1 for details). Illite dust was chosen as a well-characterized reference material previously employed in inter-comparison studies.^40^ In Fig. 2a, UnF derived from ISO experiments deviates from a log-linear relationship despite nearly identical droplet volumes implying that droplets do not have the same freezing probabilities. As in Fig. 1, this can be due to variable ISA. In Fig. 2b, the FF derived from CCR experiments increases exponentially and shows a typical sigmoidal shape.

We developed a globally optimized model based on nucleation theory for all ISO and CCR freezing experiments involving illite/ water droplets to derive the best UnF and FF representation while fitting ISA distributions (dependent on the amount of illite dust present) and *J*_het_ values (dependent only on experimental temperature). In our model, we executed 10^5^ simulations of each ISO and CCR experiment. In each simulation, we sampled the ISA per individual droplet and the number of droplets frozen at each time or temperature interval. UnF and FF were calculated for each single simulation, which is analogous to a single experiment with the same *N*_tot_. Due to random sampling of ISA, UnF, and FF for one simulation was never the same as another and, therefore, the 5th and 95th percentiles of the simulations were used for comparison with our data. The model uses the measured Brunauer–Emmett–Teller (BET) ISA (see Supplementary Table S1) as the center of the lognormal distribution and the standard deviation of the corresponding normal distribution as the width parameter (see Supplementary Table S2). We also give the surface area range that covers ±1σ. Values of the ISA distribution width in Supplementary Table S2 span 2–3 orders of magnitude, meaning that the uncertainty in surface area is about ±1 to ±1.5 orders of magnitude. This is a reasonable estimate considering the variability in illite particle number concentration per droplet, particle size distribution (PSD), settling of particles, and coagulation.

Figure 2a shows that the model captures the tendency of UnF to deviate from a log-linear relationship. In addition, observed UnF values are similar to modeled UnF and tend to fall within 5th and 95th percentiles of our predictions. Figure 2b shows that the model captures the exponential trend observed in CCR experi- ment;however, it tends to underpredict the results. One reason for this may be that the parameters in the model are very sensitive to observations of UnF in ISO experiments, which have many data points at a single temperature and thus exert a stronger constraint than CCR freezing experiments that have only one point for a given temperature. UnF and FF are cumulative and small deviations in simulated freezing initially (in the first minutes of freezing for ISO experiments or in the case of CCR experiments, at warm temperatures) propagate in time or to colder temperatures, respectively, and thus result in greater residual values between model and data. As discussed later (and in Supplementary Notes and Fig. S5), this represents a major disadvantage of the application of FF to derive nucleation kinetics. Since the actual distribution of ISA in our experiments was unknown, we repeated the model and global optimization analysis assuming a uniform distribution of ISA with a given width (Supplementary Table S2). Supplementary Fig. S6 displays these results, also demonstrating a good representation of the experimental data by the model. Hence, as long as some degree of ISA variation is considered, the model can simultaneously represent the trends in UnF and FF observed in experiments with consistent ISA distributions and *J*_het_ values.

**Fig. 3:**
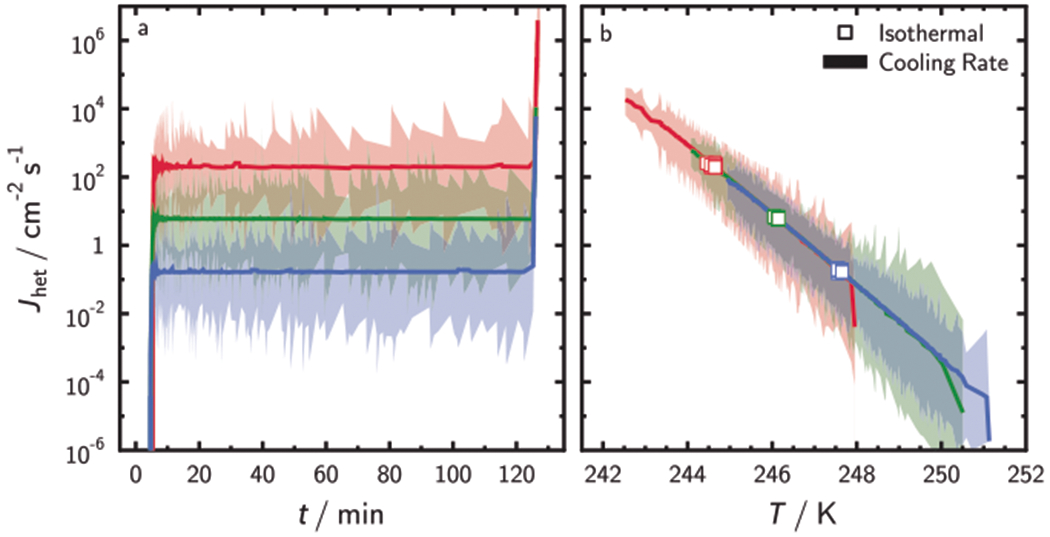
Determination of immersion freezing kinetics. Derived heterogeneous ice nucleation rate coefficients, *J*_het_, from simulation results for isothermal freezing experiments (**a**) as a function of time, *t*, and constant cooling rate freezing experiments (**b**) as a function of temperature, *T*, outlined in Supplementary Table S1. Blue color represents ISO1 and CCR1, green color represents ISO2 and CCR2, and red color represents ISO3 and CCR3. Immersed surface area per droplet is lognormally distributed (see Supplementary Table S2). Values of *J*_het_ for the isothermal experiment in **a** are shown as squares in **b** for comparison. The shadings in **a** and **b** are the upper and lower applied fiducial limits of observed freezing events for each recorded *t* or *T* interval.

Our global optimization uses a parameterization of *J*_het_ following the water activity-based immersion freezing model (ABIFM)^25,39^ with fitted constants given in Supplementary Table S2 to facilitate the description of *J*_het_ in the model. However, any other parameterization of *J*_het_ would also be applicable (see Supplementary Methods). We determined upper and lower fiducial limits of freezing from all simulations shown as shadings in Fig. 3 from Poisson statistics at *X* = 0.999 confidence^22^ to represent the stochastic uncertainty in *J*_het_ following previous work^22^ and outlined in more detail in Supplementary Methods. Figure 3a demonstrates that UnF of each ISO experiment can be represented by a constant *J*_het_ value (solid lines) in accordance with nucleation theory. ISO experiments using relatively lower ISA result in freezing at lower temperature, and, expectedly, our model concurrently yields higher *J*_het_ values. The fiducial limits shown in Fig. 3 give the stochastic uncertainty of derived *J*_het_ values but more importantly, they represent the maximum allowable range of freezing events in a given time and temperature interval, ultimately defined by the number of droplets used in the experiment (Supplementary Table S1). *N*_tot_ in all six experiments are similar and result in similar *J*_het_ uncertainty or about ±1 order of magnitude due to stochastic uncertainty.

The three CCR simulations result in *J*_het_ values that agree with each other, i.e., they overlap and follow a straight line demonstrating the exponential dependence of *J*_het_ on *T* as predicted by nucleation theory. Values of *J*_het_ from ISO experiments shown in Fig. 3a are plotted in 3b as squares and agree with those derived from CCR experiments. Assuming a uniform ISA distribution instead of a lognormal ISA distribution, does not affect these results as demonstrated in Supplementary Fig. S7. We note that an uncertainty of ±1 order of magnitude in *J*_het_ is a major improvement compared to previous IF analyses^25,40^ and this improvement is entirely achieved by using more droplets, corroborating the significant impact of stochastic freezing. When accounting for other random uncertainty, e.g., in temperature measurement, these limits can only increase because *J*_het_ is dependent on *T*. Since *J*_het_ is independent of time and surface area, its uncertainty range does not change even if the ISA distribution changes (Supplementary Fig. S7). Thus, the shading represents the limit of accuracy due to stochastic freezing for given time and temperature.

**Fig. 4:**
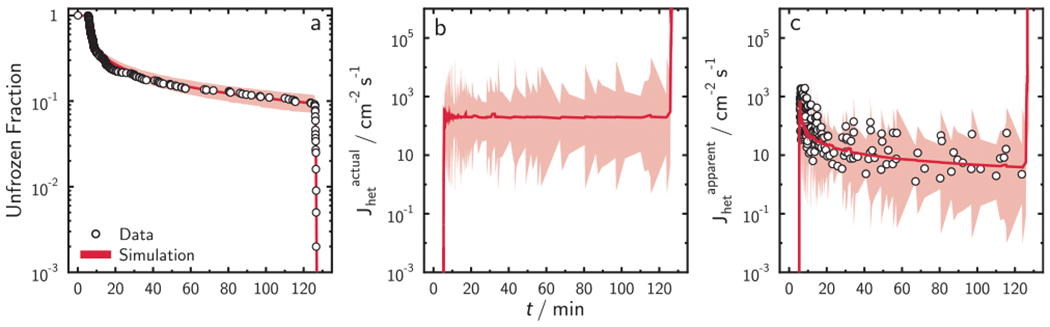
Evaluation of the assumption of identical or variable INP surface area (ISA) per droplet for isothermal freezing experiment, ISO3. **a** Experimentally derived and simulated unfrozen fraction of isothermal freezing experiment with 5th and 95th percentiles given by shading applying lognormally distributed ISA per droplet. Data in **a** are taken from Fig. 3a. **b** Calculated *J*_het_ using lognormally distributed ISA per droplet referred to as $J_{\text{het}}^{\text{actual}}$. The shading is the range of the upper and lower fiducial limits following Poission statistics at the 0.999 confidence level. Simulation results in **b** are taken from Fig. 4a. **c** Experimentally derived and recalculated *J*_het_ assuming identical ISA per droplet equal to measured mean BET values, termed $J_{\text{het}}^{\text{apparent}}$, are shown as open circles and shading, respectively. $J_{\text{het}}^{\text{apparent}}$ derived from the model was calculated from the modeled UnF in **a**. The red solid lines are mean model values.

We demonstrated that the trend of experimental UnF and FF data could be explained by a distribution of ISA and the stochastic nature of nucleation. This presents an alternative description to the concept of INAS. To provide further evidence for this approach, we perform a test (see Supplementary Notes and Fig. 4) of two assumptions that ISA in our experiments was or was not variable. First, we derive *J*_het_ using our experimental data, assuming identical ISA per droplet (Fig. 4c). Second, we apply the same simulation of UnF shown in Fig. 4a that uses lognormally distributed ISA, but make the false assumption that each droplet contains the same ISA and recalculate *J*_het_ with fiducial limits as a function of time. We term *J*_het_ using identical ISA as $J_{\text{het}}^{\text{apparent}}$ (Fig. 4c). *J*_het_ values derived from applying distributed ISA in Fig. 4b are termed $J_{\text{het}}^{\text{actual}}$ and reflect more realistic conditions.^25^ It is important to point out that experimentally derived *J*_het_ and $J_{\text{het}}^{\text{apparent}}$ are completely independent from each other and, therefore, if they are in agreement, the ISA among droplets in our experiment must have been variable.

**Fig. 5:**
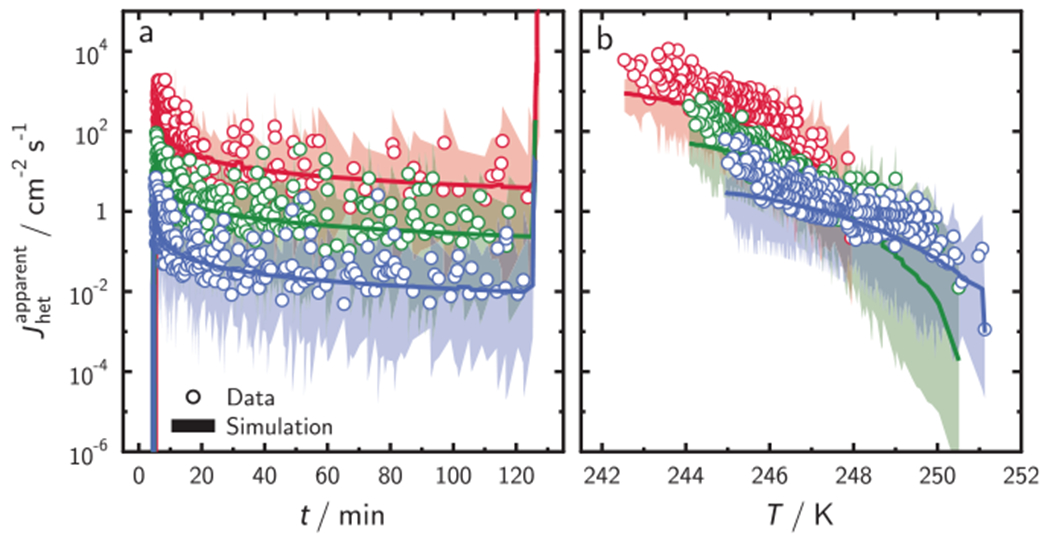
Assessment of ice-nucleating particle surface area variability postulation for immersion freezing experiments. Evaluation of the assumption of identical or variable INP surface area (ISA) per droplet for all isothermal (**a**) and constant cooling rate (**b**) immersion freezing experiments as a function of time, *t*, or temperature, *T*, respectively, given in Supplementary Table S1 for illite in water droplets. Blue color represents ISO1 and CCR1, green color represents ISO2 and CCR2, and red color represents ISO3 and CCR3. Symbols, lines, and shading are the same as in Fig. 4c. $J_{\text{het}}^{\text{apparent}}$ derived from the model in **a** and **b** was calculated from the modeled UnF in Fig. 2a and the FF in Fig. 2b with lognormally distributed ISA, however, using an incorrect assumption that droplets had identical ISA.

Figure 4c shows that the recalculated $J_{\text{het}}^{\text{apparent}}$ is in perfect agreement with experimentally derived *J*_het_, thus rejecting our false assumption of identical ISA, clearly demonstrating that assuming identical ISA per droplet is not valid. $J_{\text{het}}^{\text{apparent}}$ displays an apparent change in nucleation efficiency over time, where a high $J_{\text{het}}^{\text{apparent}}>1000\;{\text{cm}}^{-1}{\text{s}}^{-1}$ is initially observed coinciding with a large drop in UnF (Fig. 4a) while $J_{\text{het}}^{\text{apparent}}<10\;{\text{cm}}^{-1}{\text{s}}^{-1}$ at longer time. Experimentally derived *J*_het_ also exhibits apparent high and low values at the beginning and end of the ISO freezing experiment, respectively. This provides very strong evidence that not only variable ISA per droplet exists, but that the magnitude of the ISA distribution width (Supplementary Table S1) is also accurate. Since the only difference in the analyses compared to Fig. 4b is the application of identical ISA, the apparent change of *J*_het_ over time for ISO3 experiment can be attributed solely to the assumption of identical ISA. Application of a simulation that applies a uniformly distributed ISA yields the same results (Supplementary Fig. S8). We reached the same conclusion when testing our ISA variability assumptions for all ISO and CCR experiments as shown in Fig. 5 (and for the case applying a uniform distribution of ISA, Supplementary Fig. S9). This gives us confidence in our optimized ISA distribution and *J*_het_ parameters in the model for illite. We note that experimentally derived *J*_het_ datasets for CCR experiments do not overlap, but instead display an offset from each other and show a non-exponential trend with *T*. Both the non-exponential trend and offsets have been observed for other CCR experimental datasets^25^ consistent with the false assumption that droplets contained identical ISA.

As a corollary, the tests of our assumption of variable ISA also manifest the stochastic nature of nucleation when looking at the scatter of the experimentally derived and simulated data (Figs 4c and 5). The shading displays fiducial limits solely due to the stochastic nature of nucleation,^22,23^ and exactly envelope the scatter of experimentally derived Jhet values. The same results hold when conducting the evaluation of the assumptions using uniformly distributed ISA (see Supplementary Figs S8c and S9). The fiducial limits define the maximum certainty and tend to be larger than the range in the data scatter. If our experiments are repeated a greater number of times, the data scatter in *J*_het_ would still fall within these fiducial limits. This means that the variance in the data is completely explained by our stochastic model or, in other words, the scatter in *J*_het_ is entirely due to the randomness of freezing droplets.

## DISCUSSION

These analyses clearly demonstrate time-dependent and stochastic nucleation processes. These insights have several ramifications on our understanding and predictive capability of IF.

### IF studies using aliquots and droplets

Aliquot and droplet freezing experiments are usually conducted by dispensing aqueous samples on a substrate from a volume stock solution. For a sufficiently mixed colloidal suspension and perfectly generated sample or droplets (identical volumes), smaller relative uncertainties in ISA result from larger droplets. This implies that experiments using micrometer-sized droplets or sub-micrometer aerosol particles would be subject to greater ISA variability. However, typical experimental procedures result in other sources of ISA variability for stock solutions, large volume aliquots, and small droplet volumes, which include particle settling and aggregation, and non-uniform or standardized sampling. The ISA variability may exacerbate when working with ambient samples, either when washed off a substrate or after droplet formation on a substrate. Particle sizes collected on filters can span orders of magnitude. Impaction also leads to a significant size variability depending on cut-point, knowledge of particle aerodynamic diameter, and particle bounce.^41^ Therefore, solutions from collected particles inherently increases ISA variability. When condensing water to form large droplets on the substrate that is covered with ambient aerosol particles, each droplet contains an unknown number and size of particles and thus ISA will also vary significantly. It follows that if aliquots or droplets nucleate ice at some exceptional temperature, this does not necessarily mean the particles within are the most ice active. It may just result from the droplets that contain very high ISA that nucleate ice at warmer temperatures, thereby contributing ambiguity to the derived *J*_het_, *n*_s_, or INP number concentration.

### IF studies using aerosol

**Fig. 6:**
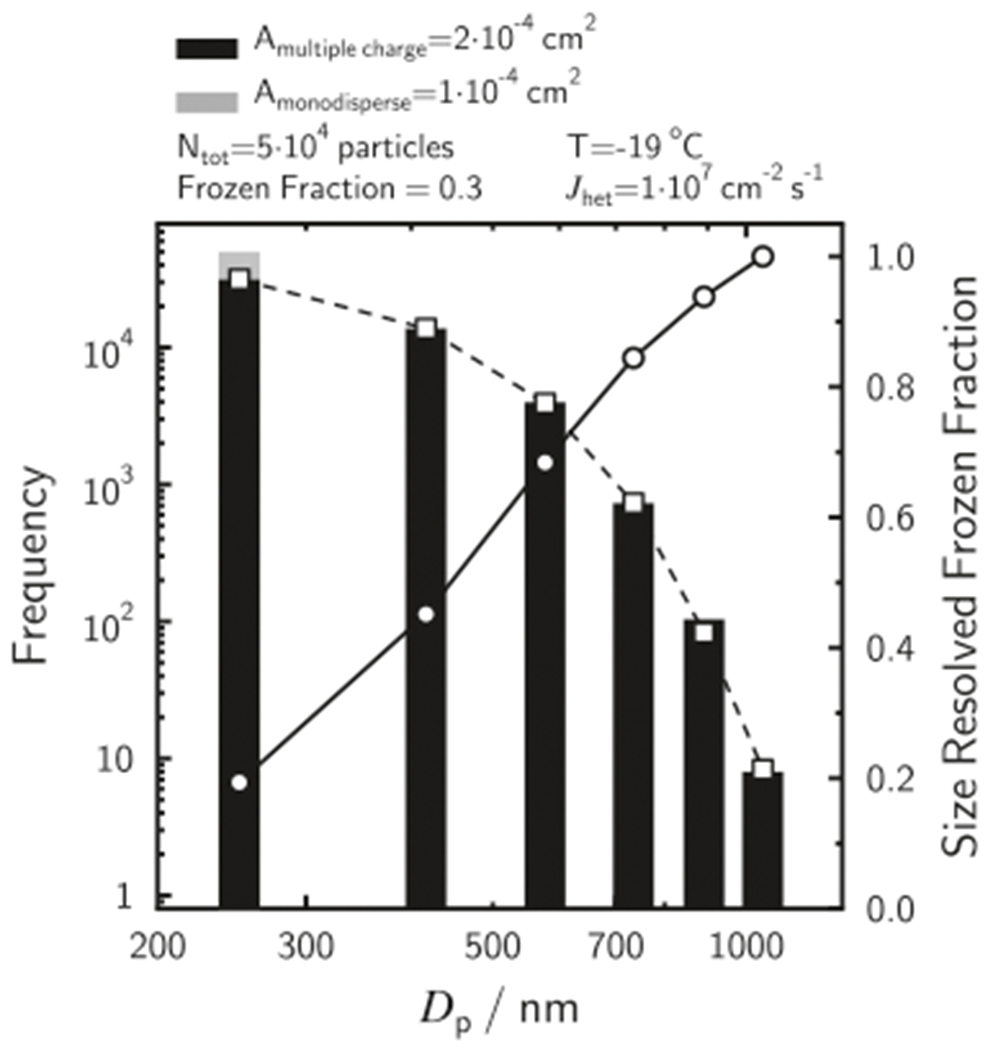
Ice-nucleating particle surface area variability when using size-selected aerosol. A total of 5 × 10^4^ particles were sampled. The expected frequency distribution is given by squares connected by dashed lines. The total particle surface area present is and 2 × 10^−4^ cm^2^ when assuming a monopolar (gray bar) and bipolar charge distribution, respectively. The size-discriminated frozen fraction was determined from the modeled frozen fraction of 0.3 with a heterogeneous ice nucleation rate coefficient, *J*_het_ = 1 × 10^7^ cm^−2^ s^−1^. The analysis applies an ice nucleation activation time of 10 s.

Selecting a monodisperse aerosol using an electrostatic particle classifier is a typical approach in ice nucleation studies.^42,43^ Fundamental to this approach is that particles are given a bipolar charge distribution, and assuming identical ISA will influence data interpretation potentially leading to incorrect conclusions^25,44^ due to aerosol multiple charging. Figure 6 shows a PSD based on the theoretical bipolar particle charge distribution^45^ when selecting particles with an electrical mobility diameter of 250 nm at 5 × 10^4^ cm^−3^. Due to multiple charging, particles larger than 1000 nm are also present and the total ISA is twice that of ISA calculated when assuming a monodisperse PSD at 250 nm. This represents an idealized case of perfectly spherical particles with the same charging efficiencies and thus represent the minimal PSD width.^46^ Of course, solid and crystalline particles are likely non-spherical, thus, enhancing PSD width. One study attempted to remove multiply charged particles with a cascade impactor before attempting to establish a bipolar equilibrium charge distribution with a charge neutralizer.^44^ The authors also measured the size distribution of particles by a different method based on the supersaturation threshold to act as a cloud condensation nuclei. When comparing the measured PSD with the expected bipolar charge distribution, the ISA distribution width was much larger than expected despite employing an impactor.^25^ Figure 6 displays an ice nucleation simulation conducted at 254.15 K with an ISA distribution (or PSD) based on the bipolar charge distribution for selected 250 nm mobility diameter particles (Fig. S10 shows the case for *T* = 258.15 K). Using *J*_het_ = 10^7^ cm^−2^ s^−1^ and an activation time of 10 s, which is typical of continuous flow diffusion chambers,^40,47^ we obtain FF = 0.3. We plot the contribution of each multiple charged particle size to the FF in Fig. 6 and demonstrate that only 20% of the 250 nm particles initiate ice formation. In other words, most of the particles with a physical diameter of 250 nm do not nucleate ice despite selecting a mobility diameter of 250 nm. The larger the particles are, the more they contribute to the observed FF. This effect is exacerbated for higher freezing temperatures (see Supplementary Fig. S10). Since FF is exponentially related to *J*_het_ or *n*_s_, inaccurate assumptions about ISA lead to substantial miscalculation and misinterpretation of nucleation kinetics. We conclude that identical particle size should not be assumed when using mobility-selected particles in a continuous flow for derivation of ice nucleation kinetics. This demonstrates that it is important to measure the actual PSD or at a minimum, to use the bi-polar charge distribution to derive the ISA per particle.

### Impact of time and stochastics on IF representation

Neglecting variations in ISA population, time dependence, and stochastic processes in nucleation can hinder the interpretation of laboratory freezing data and development of ice nucleation parameterizations that can be scaled in time and surface area from conditions in the laboratory to the atmosphere. Hence, application of Eq. (1) and equation (S1) in Supplementary Notes has to be done with great care since assuming identical ISA adds bias in the derivation of *J*_het_, *n*_s_, and similar types of parameterizations that depend on ISA. Furthermore, application of FF data to infer nucleation kinetics is prone to uncertainties. FF and UnF data represent a cumulative dataset, by definition. As such, uncertainties in single data points result in large uncertainties due to error propagation (see Supplementary Notes and Figs S5 and S6, and Fig. 2). Recent INP inter-comparison studies of IF involving up to 17 instruments indicated 2–5 orders of magnitude variations in measured *n*_s_.^40,48^ If more than one study on the same particle system uses the same assumptions, calculated *n*_s_ or *J*_het_ values would be subjected to the same bias, leading to significant misinterpretation. Accounting for ISA variability, time, and stochastic freezing with a strict uncertainty analysis can further guide experimental procedures to improve the accuracy of IF freezing kinetics such as better control of ISA and observed number of nucleation events per time interval.^25^ As an example, we demonstrate in the Supplementary Notes that our data variance is entirely explained by stochastic freezing, demonstrating strong evidence that it is the governing process of ice nucleation. Applying a time-dependent and stochastic description of ice nucleation has advantages since relevant laboratory conditions, such as time, surface area, temperature, and humidity, can be very different compared to actual cloud formation conditions in the atmosphere.^8,25^ We recommend determining *J*_het_^39,49,50^ and its uncertainty^25^ in laboratory and field studies accounting for ISA variability as demonstrated here and apply a fundamentally based parameterizations to allow for prediction of atmospheric ice nucleation with high confidence.

### The perfect ice-active particle (a “standard” INP)

To improve our understanding of the underlying processes of ice nucleation, a reference INP with defined ISA has to be developed.^51^ A “perfect” reference INP would readily provide answers about the suitability of CNT-based or singular-based approaches for describing ice nucleation. For a perfect INP, the assumption of identical ISA would be valid. Supplementary Fig. S11 shows the theoretically expected UnF and FF derived from ISO (panel a) and CCR (panel b) freezing experiments, respectively, and corresponding *J*_het_ (panels c and d). If CNT describes IF correctly, UnF from ISO experiments for a perfect INP would be log-linear and FF from CCR experiments would have a typical sigmoidal curve (“S” shape) as a function of temperature. However, as shown in Supplementary Fig. S11, stochastic ice nucleation would still induce uncertainty when a small number of droplets is used (see also Supplementary Notes).

**Fig. 7:**
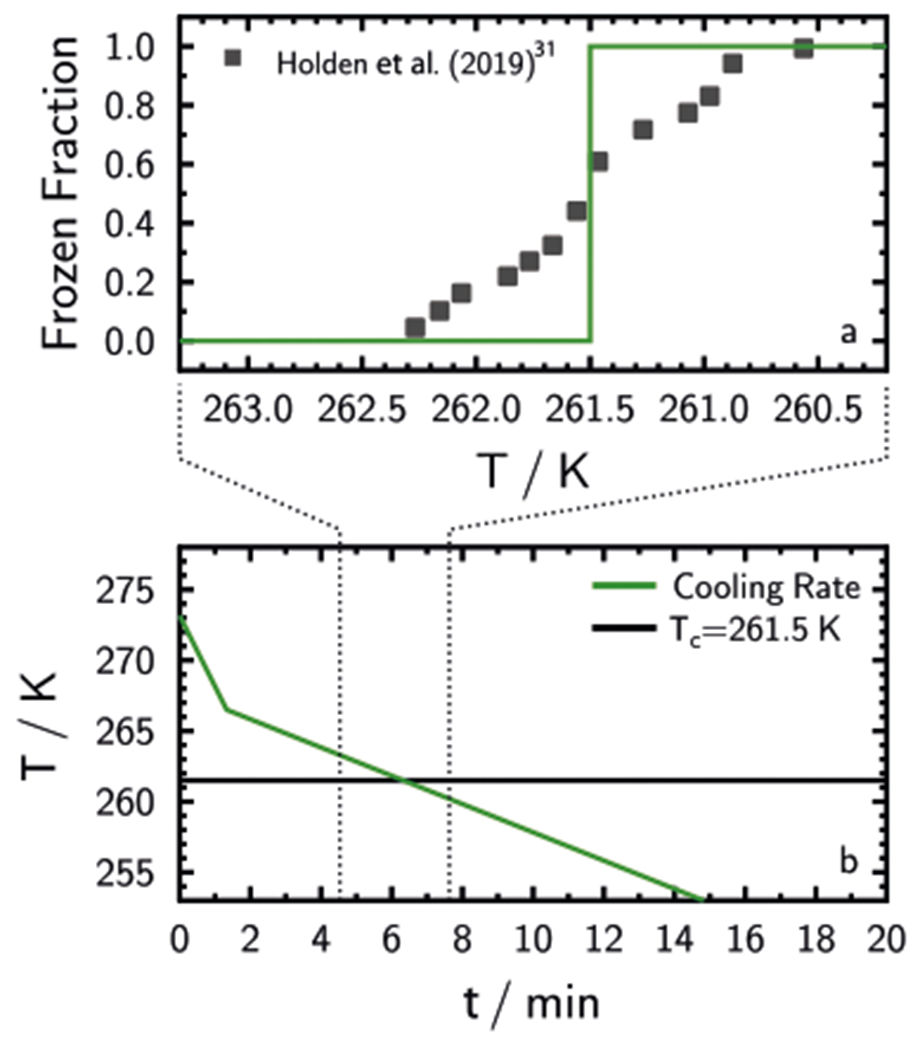
Example experiment of a perfect ice-active particle population following the time-independent description. The green line in **a** and **b** shows the frozen fraction for a cooling rate experiment and the experimental temperature, *T*, profile over time, *t*, respectively. The characteristic temperature at which all ice-active sites on particles nucleate ice, *T*_c_, is indicated. Note that **b** is enlarged to show a longer time period. Squares in **a** show the frozen fraction for a single nucleation site.^31^

Following the singular *n*_s_ approach, a perfect ice-active particle would have sites that all activate at an identical characteristic temperature, *T*_c_. Freezing for *T* > *T*_c_ would be impossible as shown in the CCR simulation in Fig. 7 for a perfect INP with *T*_c_ = 261.5 K. In Supplementary Fig. S12, we show that FF and UnF for CCR and ISO experiments of a single INP with *T*_c_ = 261.5 K are very different from our current experimental findings.

The question remains, do well-defined, favorable ice-active sites exist on INPs and if found, would one of them always nucleate ice at the same temperature? Recently, Holden et al.^31^ used highspeed optical microscopy to identify ice nucleation on solid substrates in contact with millimeter-sized droplets and observed that crystallization occurred mostly in the same spot for sequential freezing and melting cycles. Using scanning electron microscopy and atomic force microscopy, they found that micrometer-sized morphological defects were present where ice nucleation occurred.^31^ We follow the authors and interpret this single defect as hosting a single ice-active site. The measured FF of a single site^31^ is plotted in Fig. 7a as black squares. Notice that even a single active site (as defined in that study) does not instantaneously trigger freezing at some *T*_c_ but deviates around 2 K. The authors suggest that there is some freezing variability around *T*_c_, however, this is only a phenomenological description without yet a physical basis.^52^ Therefore, the FF of this single site is more in line with a time-dependent and stochastic freezing process.

We presented an IF interpretation that unambiguously demonstrates that the nucleation kinetics analysis has to consider the stochastic nature of freezing and varying ISA among droplets. The insight gained from our analysis challenges several aspects commonly employed in IF studies, including its mathematical description and the lack of a time-dependent freezing process. All of our data scatter is explained by stochastic freezing and a J_he_t parameter as a function only of temperature. This finding challenges the role of active sites and the application of a distribution of active sites with varying ice formation potential to interpret the freezing trends in heterogeneous ice nucleation experiments. We also challenge future heterogeneous ice nuclea- tion studies to strictly characterize their data variance and encourage its quantitative explanation. The major advantage of this approach is that it depends on a rigorous uncertainty analysis of the ice nucleating surface area, ice nucleation statistics and quantifying the nanoscale-characterization of INP surfaces, all of which can be (in principle) directly measured. Doing so will greatly enhance the understanding of ice nucleation and move into new and practical directions to improve prediction of ice nucleation using a fundamental theory, developing new theoretical extensions based on physical parameters as done in previous literature,^25,39,53–57^ and foster new technological innovation in surface science and engineering. We expect that our results also hold when analyzing nucleation from any supercooled liquid when in contact with a nucleating substrate, thereby greatly extending the applicability and falsifiability of our proposed approach.

## METHODS

### WeIzmann supercooled droplets observation on a microarray

The WeIzmann supercooled droplets observation on a microarray (WISDOM) is a microfluidic device based on the design of Schmitz et al.^58^ The WISDOM setup and validation are described in Reicher et al.^59^ Supplementary Fig. S13 presents custom-built microfluidic chip with droplets ~100 μm in diameter attached to a microscope slide and the WISDOM setup. Droplet generation was achieved by injecting the sample solution (deionized water, DIW, or illite-NX suspended in DIW) and oil (98% mineral oil and 2% Span 80 mixture) into the microfluidic device using pneumatic pumps (NE-500 Programmable OEM Syringe Pump rate). Prior to the droplet generation process, the sampled suspensions were thoroughly mixed and sonicated three times for 40 s (Hielscher, UP200st). The sample flows perpendicular to the oil, resulting in sheering off individual droplets that continue to move into the channel (Supplementary Fig. S13). Changing the oil and/or the sample flows, allows us to control the droplet size. Once the droplets are generated and arranged in an array, the microfluidic device is placed on a cooling stage (Linkam LTS420) attached to an optical microscope (Olympus BX51). Freezing experiments are monitored using a CCD camera. By following the immediate changes in the gray scale level of a freezing droplet, the freezing events are detected automatically for each individual droplet. A temperature calibration was applied by detection of the liquefaction and eutectic temperatures of solutions with different water activity as described by Reicher et al.^59^ and Zipori et al.^16^

### Constant cooling rate experiments and isothermal experiments

We performed two types of experiments: CCR and ISO freezing experiments. In CCR experiments, the droplets were cooled at 20 K min^−1^ to ~8 K higher than the expected start of the droplets’ freezing. Subsequently, a slower cooling rate of 1 K min^−1^ was applied to monitor the individual droplet freezing events. After freezing, the droplets were heated and the ice melting point was measured at a heating rate of 1 K min^−1^. In ISO freezing experiments, the droplets were cooled from 273.15 at 5 K min^−1^ to the temperature at which ~10% of the drops froze. From this point on, the temperature was held constant for 2 h. For direct comparison of nucleation data, we conducted both CCR and ISO freezing experiments with the same droplet samples.

### IF simulations

The model used here, based on Alpert and Knopf,^25^ is a statistical Monte Carlo model made up of numerous simulations. Nucleation is represented by binomial statistics following Koop et al.^22^ Model input includes the number of droplets, ISA, and *J*_het_ as a function of temperature parameterized following ABIFM.^39^ Modeled UnF or FF were fitted to all datasets globally with five optimizable parameters, three for ISA distribution width and two for the ABIFM^39^ shown in Supplementary Table S2. The freezing probability for a single droplet at any time is derived from *J*_het_, the droplet ISA, and the simulation time, which is equal to the observed nucleation time. At any given time step, the freezing probability is used in a binomial random sample. If freezing is registered, the frozen droplet is not considered in subsequent time steps. A Monte Carlo method of 10^5^ simulations is used for a complete model run, each initialized with a random sample from ISA distributions, which are either lognormal, uniform, or a Dirac. The latter is when identical ISA per droplet was used. Mean values of UnF and FF with 5th and 95th percentiles are derived. A record of ISA in every droplet and the time and temperature that droplet froze is kept, thereby allowing the mean *J*_het_ and upper and lower fiducial limits following Poisson statistics to be derived.

### Materials

In this study, we use illite-NX^60,61^ in a 5 wt% aqueous stock suspension. The stock solution was diluted to yield examined suspension concentrations of 1 wt%, 0.1 wt%, and 0.005 wt%. After preparation of the illite/water stock solution, it was stirred for 24 h. We performed all freezing experiments within 8 days from the time of the fresh stock solution preparation.

## Supplementary Material

Supplement

## Figures and Tables

**Table 1.** Nomenclature.

CNT	Classical nucleation theory
INAS	Ice nucleation active sites
INP	Ice-nucleating particle
IF	Immersion freezing
ISO	Isothermal immersion freezing experiments
CCR	Constant cooling rate immersion freezing experiments
FF	Frozen fraction of droplets
UnF	Unfrozen fraction of droplets
*n*_s_	Ice-active sites number density
ISA	Ice-nucleating particle surface area
*J*_het_	Heterogeneous ice nucleation rate coefficient
$J_{\text{het}}^{\text{actual}}$	Heterogeneous ice nucleation rate coefficient applying distributed ISA
$J_{\text{het}}^{\text{apparent}}$	Heterogeneous ice nucleation rate coefficient applying identical ISA
