# Supplementary material for: Stochastic nucleation processes and substrate abundance explain time-dependent freezing in supercooled droplets
Source: NPJ Clim Atmos Sci. Author manuscript; Available in PMC 2020 Aug 4. (PMC7402410; doi:10.1038/s41612-020-0106-4)
Supplement: Supplement [file NIHMS1612874-supplement-Supplement.pdf]

Supplementary Information

Stochastic nucleation processes and substrate abundance explain time-dependent freezing in supercooled droplets

Daniel A. Knopf[1]*, Peter A. Alpert[2], Assaf Zipori[3], Naama Reicher[3], Yinon Rudich[3]*

[1]Institute for Terrestrial and Planetary Atmospheres, School of Marine and Atmospheric Sciences, Stony Brook University, Stony Brook 11794-5000, New York, USA

[2]Laboratory of Environmental Chemistry, Paul Scherrer Institute, 5232 Villigen, Switzerland.

[3]Department of Earth and Planetary Sciences, Weizmann Institute of Science, Rehovot 76100 Israel.

*Corresponding authors: daniel.knopf@stonybrook.edu and yinon.rudich@weizmann.ac.il

Supplementary Notes

Applied Concepts, Theories, and Definitions of Terms

Ice-Active Sites. *In situ* determination of ice nucleation of an active site has not yet been achieved[1]. *In situ* observations at least on a single-nanometer resolution are needed since the critical nuclei are just nanometers in size. Observing immersion freezing on the nanometer scale is even more challenging since detection of active sites has to be conducted in the presence of a macroscopic layer of water, something that is not currently feasible. Computational simulations also do not provide a clear picture of the underlying physicochemical properties of an active ice nucleation site[1]. It is known that ice does not form at the surface of a substrate, but 1-3 monolayers away thus further complicating the definition of an active site[1]. Therefore, attributing an observed spread in frozen fraction (*FF*) and unfrozen fraction (*UnF*) curves to different types of actives sites on the same material in various droplets, remains only a concept.

To avoid this ambiguous approach for the interpretation of immersion freezing data and to promote the use of testable parameters, we assume that increasing the surface area of the ice nucleating material exponentially increases the probability to nucleate ice. This assumption is rooted in nucleation theory[2]. However, we assume that nucleation among droplets is initiated by the same dominating surface features, those that make one material a better INP compared to another material. In accord with nucleation theory, we also dictate that the nucleation initiated by these surface features occurs randomly[2,3].

In this study, we challenge the time-independent deterministic property inherent in the use of ice nucleation active site (INAS) densities, n_s, in units of cm^{-2}. It assumes that each nucleation event occurs on a single ice active site. n_s is a function of temperature only and it describes the number of ice nucleation events per surface area at a given temperature. For now, it is a mathematical construct and, in fact, does not reflect the presence of ice active sites, but gives the number of nucleation events that can happen anywhere on 1 cm^2 of ISA. It does not allow ice nucleation to proceed as time passes at a fixed temperature. The fact that n_s accounts only for the temperature dependence is the fundamental reason that this description is termed

singular or deterministic. As a direct consequence of this concept, freezing occurring as a function of temperature invokes the presence of different types of actives sites to explain the data despite being the same overall particle-type. This study is directed at this time independent, ice-active sites view (n_s-based approach) which is commonly used for presentation and interpretation of freezing data.

The interpretation that is presented is based on nucleation theory[2,3]. It is well established that the formation of a critical ice nucleus is fundamentally a random process. This is because the fluxes of molecules from the evaporating or to the growing nucleus are defined by diffusion which is inherently random. Also, the probability of an ice nucleus formation among the many water molecules is very small. Since the fluxes of molecules define the formation of the critical nucleus, nucleation is also inherently time-dependent. Thus, nucleation theory provides a stochastic and time dependent description of ice nucleation as its basic axioms allowing derivation of the heterogeneous ice nucleation coefficient (J_{het}). J_{het} is second order in nature to capture the time and surface area dependence (units $\mathrm{cm^{-2}\ s^{-1}}$) of the nucleation rate (units $\mathrm{s^{-1}}$)[2-4] and depends only on temperature. This theoretical parameter is a material-specific, meaning it can be different for different materials under the same thermodynamic conditions.

Isothermal Freezing Experiments and Ice-Active Site Density

Figure S1 shows the expected uncertainty of the time-independent ice active surface site density, n_{s}, when derived from unfrozen droplet fraction (*UnF*) curves obtained from isothermal freezing experiments shown in Fig. 1b. n_{s} is derived for initial and final *UnF* according to

$$\frac{N_{\mathrm{liq}}}{N_{\mathrm{tot}}} = e^{-n_{\mathrm{s}}A}, \qquad \text{(S1)}$$

where N_{tot} is the total number of liquid droplets at the beginning of the experiment, N_{liq} is the number of droplets that remain liquid as a function of temperature and A is the ice nucleating particle surface area, ISA, in each droplet. Several orders of magnitude of uncertainty in n_{s} exists simply due to it being time independent for isothermal experiments. It is interesting to note that this uncertainty range becomes smaller when *UnF* does not decrease by much. If, e.g., only 1% of the droplets froze during the isothermal

temperature period, then the ISA at the beginning of the experiment would differ by only 1%. It follows that the n_s error due to neglecting time would be biased by 1%. However, this is misleading because when only a few freezing events occur, there should be great uncertainty due to poor statistics not reflected in the range of *UnF* values.

Figure S4 shows the case of constant cooling rate immersion freezing experiments for ISA conditions presented in Figs. 1 and S1. Figure S4a displays exemplary temperature and J_{het} profiles and resulting frozen droplet fractions (*FF*) are shown in (b). As demonstrated in Fig. S4c, for constant cooling rate experiments, n_s is log-linear when identical ISA is used and curved when variable ISA is used. This is consistent with isothermal experiments.

Testing the Assumption of Variable ISA

To test the validity of applying varying ISA, we perform an experiment opposite from our approach, i.e., assuming that the ISA per droplet in the experiments is actually identical or close enough to be reasonably approximated as identical. Rejection of this assumption implies accepting that the ISA in the droplets has large variations. Figure 4 visualizes this exercise for the ISO3 dataset (Table S1), where Figs. 4a and b are reproduced from Figs. 2a and 3a, respectively. We first derive J_{het} using our experimental data only (without any model parameters) following previous analyses[5-9], using the number of observed freezing events and time under the assumption of identical ISA per droplet equal to the mean BET surface area. These experimentally derived J_{het} are shown as circles in Fig. 4c. Second, we apply the same simulation of *UnF* shown in Fig. 4a that uses lognormally distributed ISA but make the false assumption that each droplet contains the same ISA and recalculate J_{het} as a function of time. In other words, we compare the incorrectly calculated J_{het} from the model of ISO3 assuming identical ISA with experimental J_{het} data derived using identical ISA. The recalculated J_{het} under the false assumption of identical ISA with fiducial limits are shown as the red line and shading in Fig. 4c, respectively. We term J_{het} values derived from applying distributed ISA in the model in Fig. 4b as J_{het}^{actual}. This emphasizes the application of a distribution of ISA reflecting more realistic conditions[10]. Derivations of J_{het} using identical ISA are termed $J_{het}^{apparent}$ (Fig. 4c).

It is important to point out that experimentally derived J_{het} and $J_{\mathrm{het}}^{\mathrm{apparent}}$ are completely independent from each other and therefore, they must agree to reject our false assumption. We reiterate that if rejected, ISA among droplets in our experiment must have been variable.

Uncertainty of Heterogeneous Ice Nucleation Rate Coefficients and Ice Nucleation Active Site Densities from Frozen Fraction Derivations

We have made calculations of the standard error on J_{het} and n_{s}, σ_{Jhet} and σ_{ns}, respectively, to illustrate the implications of propogating errors using frozen fractions without any concern for stochastic uncertainty due to the number of observed freezing events (Fig. S5). The familiar equations for frozen fraction are rearranged in the form

$$J_{\mathrm{het}} = \frac{-\ln(1-FF)}{At} \tag{S2}$$

and

$$n_{\mathrm{s}} = \frac{-\ln(1-FF)}{A}\,. \tag{S3}$$

Error propagation through quadrature is used to derive the following relationships

$$\left(\frac{\sigma_{J_{\mathrm{het}}}}{J_{\mathrm{het}}}\right)^2 = \left(\frac{\sigma_{n_{\mathrm{s}}}}{n_{\mathrm{s}}}\right)^2 = \left(\frac{\sigma_A}{A}\right)^2 + \left(\frac{\sigma_{FF}}{(1-FF)\ln(1-FF)}\right)^2\,. \tag{S4}$$

We use an error of σ_A/A=20% roughly equivalent to 20% error on droplet volume and 6% error on droplet diameter. The latter is the same error on droplet diameter reported here. The error in *FF* data points was chosen to be about ±0.1, which is representative of the 5th and 95th percentiles from simulations in this study.

In any CCR experiment, the very first and very last freezing events that occur are the most uncertain because only a small number of droplets freeze when temperatures are high, and a small number of droplets remain to be frozen at the end of the experiment when the temperatures are low. The error in J_{het} and n_{s} in Fig. S5b and c, respectively, initially is very small at warm temperature and increases drastically at colder

temperatures. This is opposite to what is expected, and therefore the uncertainty in *FF* assuming identical ISA is argued here to be a poor indicator of experimental uncertainty when deriving J_{het} and n_s. More correctly, J_{het} at the beginning and end of CCR experiments have the largest spread despite different droplet numbers.

The uncertainty in *FF* and *UnF* due to freezing at the beginning of experiments cascades to later times resulting in a compounded uncertainty. To optimize our model parameters, the average *UnF* or *FF* from 10^5 simulations was fit to *UnF* or *FF* from 6 experiments. Average *UnF* or *FF* from our model had a smooth shape and defined trajectory, while a single experiment had steps and discontinuities due to data scatter. This will result in our model fit to be systematically worse, i.e., have larger residual values, over time. This contributes to the residuals between experimentally derived and simulated *UnF* and *FF* in Figs. 2 and S6. As discussed in the main text, the residual values for *FF* appear greater than for *UnF*, but this is likely due to the ISO experiments that tightly constrain the model at the three investigated temperatures. Notice that in all experiments, the agreement between observed and modeled *UnF* and *FF* is not perfect, but there is excellent agreement for experimentally and model derived J_{het} as seen in Figs. 4, 5, S8 and S9. As argued in the main text, this satisfies our assumption of variable ISA, however, it also shows that all freezing data are still within the stochastic uncertainty (fiducial limits). Although assuming identical ISA makes for a straightforward mathematical derivation of *FF*, it is strongly discouraged to use this approach as it leads to misrepresentation of not only J_{het} and n_s, but their uncertainty as well. As an alternative, we recommend determining J_{het} and its uncertainty without the use of frozen or unfrozen fractions. Following Zobrist et al.[6] and Alpert et al.[11] the ISA per droplet should be accounted for, then used together with the number of nucleation events in a measured time interval (not the fraction out of the total) to calculate the nucleation rate coefficient. When applying a representative stochastic uncertainty of ±1 order of magnitude in J_{het}, simulations (not shown) and experimental data of *UnF* and *FF* (Figs. 2 and S6) show significantly better agreement than 5th and 95th percentiles.

Explained and unexplained variance in experimental data sets

The test of our assumption of the presence of variable ISA, allows for a unique comparison of the fiducial limits and the variance of J_{het} data. The data variance can be expressed, as the variance that can be explained by the model and the variance that is unexplained. Our model variance is entirely due to random freezing behavior. Any other unexplained variance may be due to, e.g., temperature error, error in the mean BET surface area, but could also be due to the ice active sites that were not included in the model. We define the data variance, v_{data}, on a log scale as

$$v_{\mathrm{data}} = \frac{\sum_{i=1}^{N}\left(\log J_{\mathrm{het},i}^{\mathrm{apparent}} - \log E\left(J_{\mathrm{het}}^{\mathrm{apparent}}\right)\right)^2}{N_{\mathrm{tot}}-1}, \quad \text{(S5)}$$

where $J_{\mathrm{het},i}^{\mathrm{apparent}}$ is the value of a data point and $E\left(J_{\mathrm{het}}^{\mathrm{apparent}}\right)$ is the expected value for the same temperature and time. Using Eq. (S5), v_{data}= 0.3 for all experimental data, i.e., a standard deviation of ±0.5 orders of magnitude. The majority of data scatter in Figs. 5 and S9 is within the range of the fiducial limits which deviate about ±1 order of magnitude from $E\left(J_{\mathrm{het}}^{\mathrm{apparent}}\right)$. This demonstrates that the data variance can be entirely explained by stochastic freezing. It follows that any unexplained variance, e.g., by ignoring any quantification of ice active sites, must be minor compared to stochastic freezing. This finding suggests that stochastic freezing is the main cause of observed data scatter in the presented experiments, and thus the governing process of ice nucleation.

Sensitivity of Frozen Fractions and Heterogeneous Ice Nucleation Rate Coefficients on Droplet Numbers

We performed simulations of isothermal and cooling rate experiments to demonstrate the effect that stochastic uncertainty has on unfrozen fraction, *UnF*, frozen fraction, *FF*, and heterogeneous ice nucleation rate coefficients, J_{het}, when 30, 100, and 1000 droplets are used but assuming droplets have identical ISA. Figure S11 shows that when using more droplets in both types of simulations, the 5th and 95th percentiles ranges shown as shading are more narrow (blue) compared to using less droplets (green and red). Upper and lower fiducial limits of J_{het} behave similarly, that more droplets lead to less uncertainty. A minimum

uncertainty always occurs when most of the droplets freeze, typically close to median *FF* values and grows larger at the beginning and end of experiments. For example, using 100 droplets in CCR experiments in Fig. S11d, the range of J_{het} is 4 orders of magnitude at the lowest temperature, shrinking to 1 order of magnitude at its waist, and up to 6 or more orders of magnitude at warmer temperatures. Likewise for ISO experiments in Fig. S11c, the uncertainty is initally large as temperature is rapidly decreased from 273.15 K, then becomes small during the isothermal time interval and grows large again as the experiment finishes.

We note the uncertainty definitions on *UnF*, *FF*, and J_{het} are not the same. For $t > 15$ min, the *UnF* falls to $<10^{-1}$ meaning that only <10% of droplets remain (Fig. S11a). There are far too few droplets left over to freeze at later times resulting in significant uncertainty. This is reflectled by the range of values increasing as time increases from 15 min. However, *UnF* at $t < 6$ min are very small indicating very little error; however, this is not the case due to very few freezing events at this time. The limits in J_{het} are much wider and demonstrate the actual uncertainty. Therefore, we caution readers when using *UnF* or *FF* values to make claims about ice nucleation uncertainty or any sensitivity of freezing on parameters such as time, surface area, or temperature. Using uncertainties of *UnF* or *FF* without considering stochastic error can result in incorrect interpretations. Instead, we suggest the use of nucleation rates or nucleation rate coefficients (i.e., ω_{het} and J_{het}) and fiducial limits of Poisson statistics as uncertainty estimates.

Ice Nucleation Due to Perfect Particles Having Identical Ice Active Sites

Figure S12 presents *UnF* and *FF* for a population of particles all having ice active sites that trigger freezing at an identical characteristic temperature, $T_c = 261.5$ K. For such a particle population, when $T < T_c$ then *FF* = 1 and *UnF* = 0. We show 2 ISO and 1 CCR experimental temperature trajectories. Figure S12a shows that when cooling from 273.15 K to a desired temperature, T_{hold}, which is then held constant over time, only when $T < T_c$ is freezing observed. When $T_{hold} < T_c$, freezing occurs instantaneously during the experimental cooling ramp prior to T_{hold} is reached.

Supplementary Methods

Homogeneous Ice Nucleation

Using WISDOM, shown in Fig. S13, we measured homogeneous ice nucleation rate coefficients (J_{hom}) of pure water droplets (Fig. S2, Table S1). Isothermal and constant cooling rate homogeneous freezing experiments of water droplets have also been previously reported using a microfluidics ice nucleation setup WISDOM[12] (Fig. S2). Nearly monodisperse water droplets (mean diameter = 85.5 μm, σ=5 μm) were employed in our experiments (Table S1). Thus, we do not expect large variations in homogenous freezing probabilities due to differences in droplet volumes[2,3]. As expected, we observed a very narrow freezing distribution with *T* when conducting constant cooling rate freezing experiments (Fig. S2a), in agreement with previous work[12-14]. Also, derived J_{hom} values fall in the range of reported values[12-19] (Fig. S2b). Figure S3 shows the results of isothermal homogeneous freezing experiments, displaying the CNT-predicted exponential behavior of the *UnF* with time. In summary, when monitoring hundreds of isothermal freezing events to reduce the scatter in the *UnF* data due to the stochastic nature of the freezing process[2,3,10] and applying monodisperse droplets, *UnF* displays the expected log-linear behavior with time.

Global Optimization: Parameterization of J_{het}

Our global optimization uses a parameterization of J_{het} following the water activity-based immersion freezing model (ABIFM)[8,10] with fitted constants given in Table S2. In turn, J_{het} is then used to calculate the probability that a single droplet will freeze. Application of ABIFM has only the purpose to facilitate the description of J_{het} in the model, however, any other parameterization of J_{het} would also be applicable. We evaluate the uncertainty of J_{het} for illite using Monte Carlo simulations. Although J_{het} is parameterized identically in every simulation to calculate freezing probability, random sampling causes nucleation events at different times and in droplets with different ISA. Therefore, we can recalculate J_{het} as a function of

temperature for each of the 10^5 simulations as done with modeled *UnF* and *FF*. As before, the recalculated J_{het} from one simulation will not be the same as from another.

Fiducial Limits of Ice Nucleation

Since nucleation is fundamentally a stochastic process[2,3], it can be expressed by a binomial probability distribution as discussed in detail elsewhere[3] and only briefly summarized here. Following Koop et al. (1997)[3], the nucleation rate ω can be expressed as

$$\omega = \frac{N_{\mathrm{nuc}}}{t}, \qquad \text{(S6)}$$

where t is the nucleation time and N_{nuc} is the number of nucleation events. The underlying statistical nature of nucleation can then be used to derive a statistical uncertainty in ω for a given number of detected nucleation events on a fixed confidence level, χ=0.999. Thus, Eq. (S6) yields the most likely value for ω under arbitrary experimental conditions. The lower fiducial limit, ω_{low}, is defined such that less than N_{nuc} nucleation events would occur with a probability x, if ω_{low} were the true nucleation rate. Even if no single nucleation event occurs (N_{nuc} = 0), an upper fiducial limit for ω can be determined. As given in Table 2 of Appendix 2 in Koop et al. (1997)[3], when N_{nuc} = 1, its upper fiducial limit is 9.233 and its lower fiducial limit is 0.001 nucleation events, spanning almost 4 orders of magnitude in uncertainty. As the number of N_{nuc} increases, the fiducial limits become much tighter since the nucleation statistics improve.

Supplementary Figures

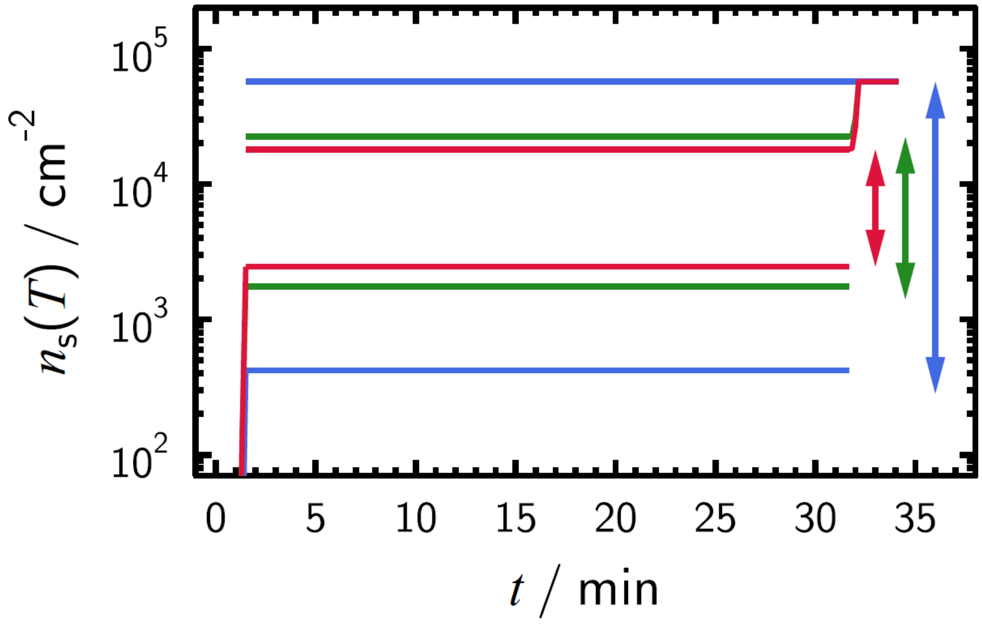

Figure S1| Isothermal immersion freezing experiments interpreted using the ice active surface site density n_s(T). Uncertainty of time-independent n_s(T) when derived from unfrozen fraction curves shown in Fig. 1b. Blue, green and red arrows mark the range of n_s uncertainty for water droplets containing identical surface area and lognormally and uniformly distributed ice nucleating particle surface area (ISA) immersed in droplets, respectively.

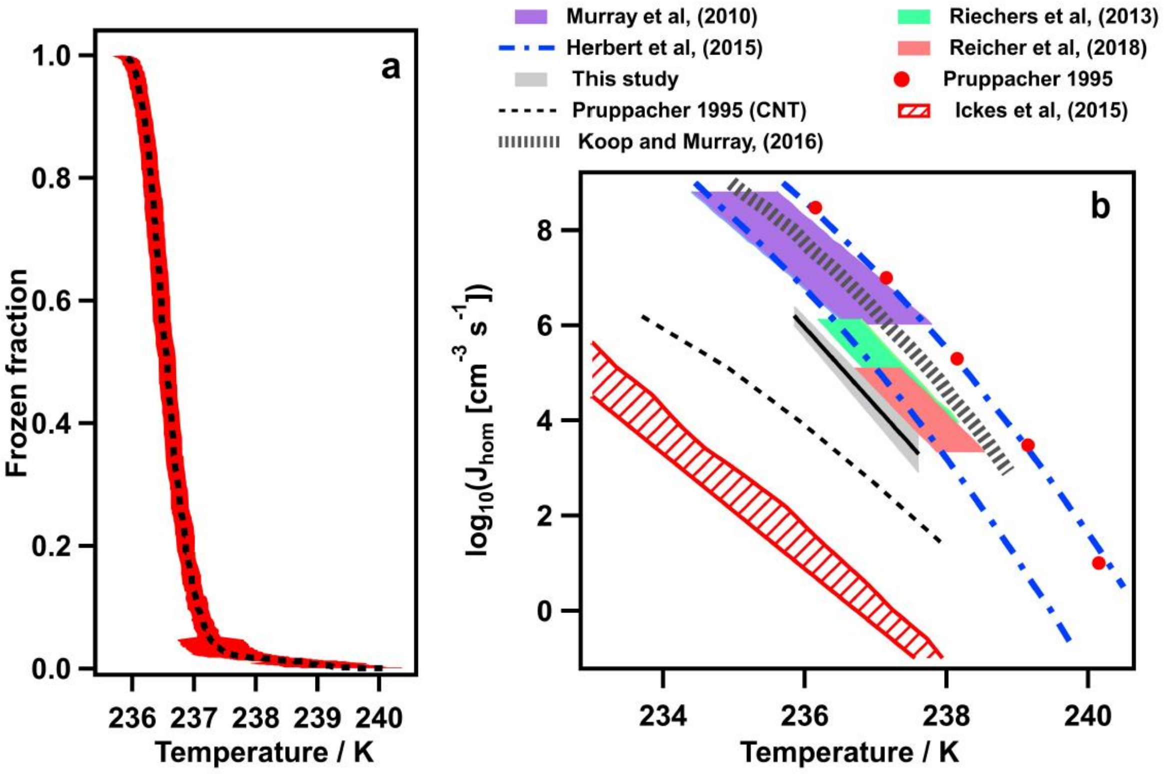

Figure S2| Ice nucleation in deionized water droplets. (a) Water droplet freezing as derived from constant cooling experiments using WISDOM (see Table S1). The black dashed line is the mean frozen fraction and the shaded red area represents one standard deviation. (b) Derived J_{hom}(T) from (a) in comparison with data by Murray *et al*. (2010)[14], Pruppacher (1995)[17], Herbert *et al*. (2015)[19], Riechers *et al*. (2013)[13], Reicher *et al*. (2018)[12], Ickes *et al*. (2015)[16], and Koop and Murray (2016)[18].

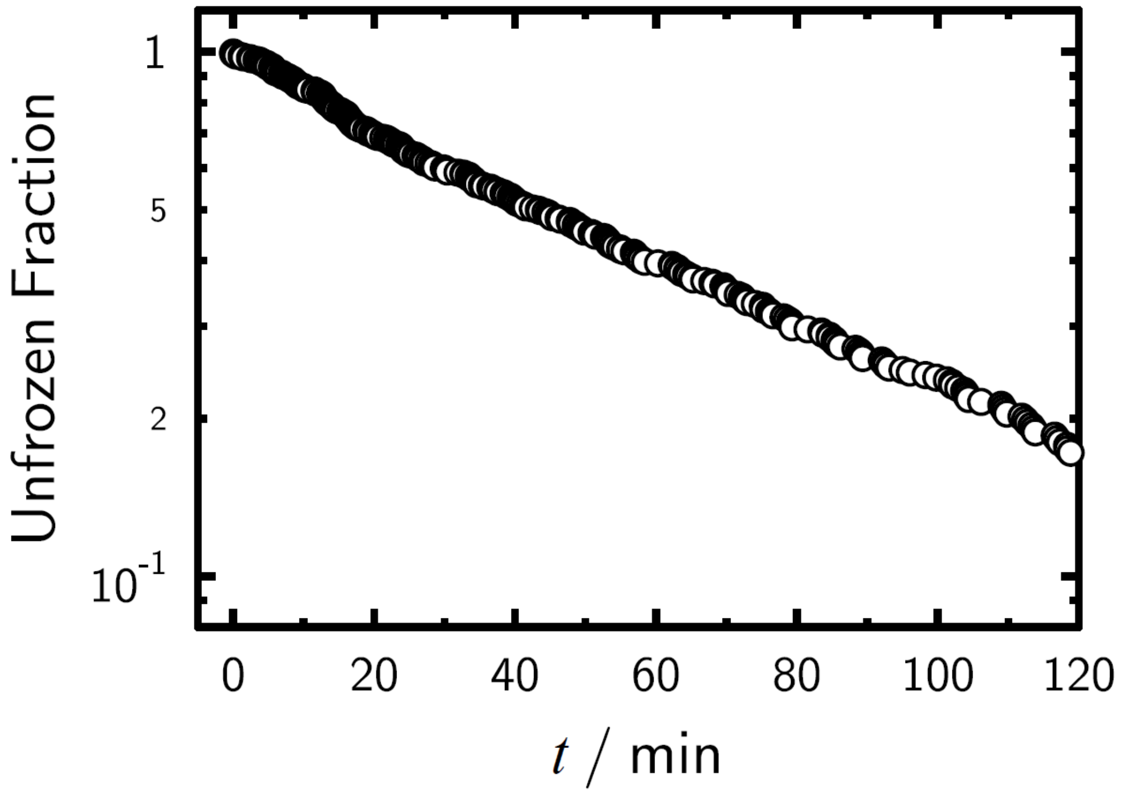

Figure S3| Isothermal freezing of 383 water droplets, 85.5 µm in diameter (σ=±5 µm), held at 237.15 K as a function of time, *t*.

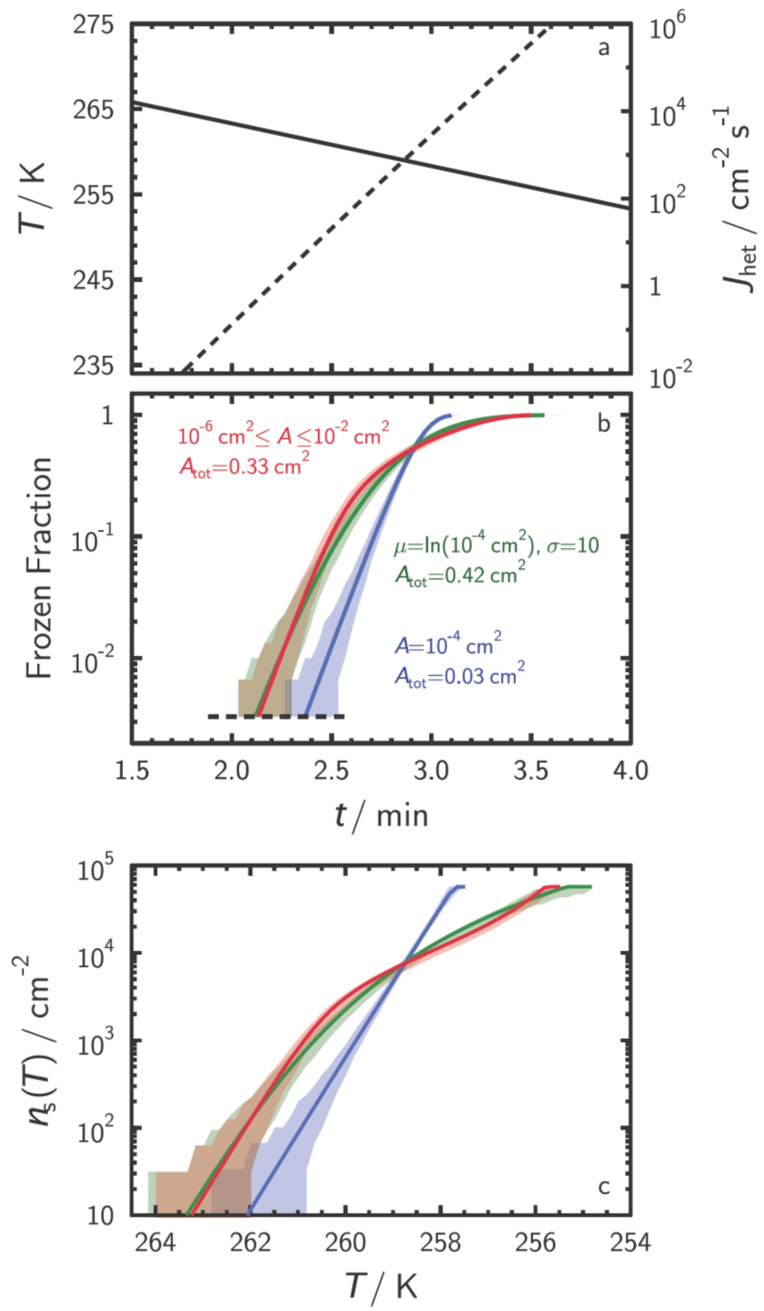

Figure S4| Constant cooling rate (CCR) immersion freezing experiment. (a) A typical experimental temperature, T, profile over time, t, (solid line) where the droplets are cooled at 5 K min^{-1}. Dashed line shows an example of the corresponding heterogeneous ice nucleation rate coefficient, J_{het}. (b) Exemplary profile of the frozen droplet fraction as a function of t derived from simulating freezing using 300 droplets. All curves apply the same J_{het} depicted in (a). Shaded areas represent 5th and 95th percentiles[3,10]. Total surface area, A_{tot}, is indicated. (c) Ice active surface site density, $n_s(T)$, derived from the frozen fractions in panel (b). Uncertainty of time-independent $n_s(T)$ is calculated from the percentile bounds of frozen fraction. The blue solid lines represent simulated frozen droplet fraction assuming identical ice nucleating particle surface area, ISA, of $A=10^{-4}$ cm^2 in each droplet. The green lines represent the case of a lognormally distributed ISA around A, with σ = 10. The red lines represent the case where ISA is uniformly distributed by ±2 orders of magnitude in ISA around A.

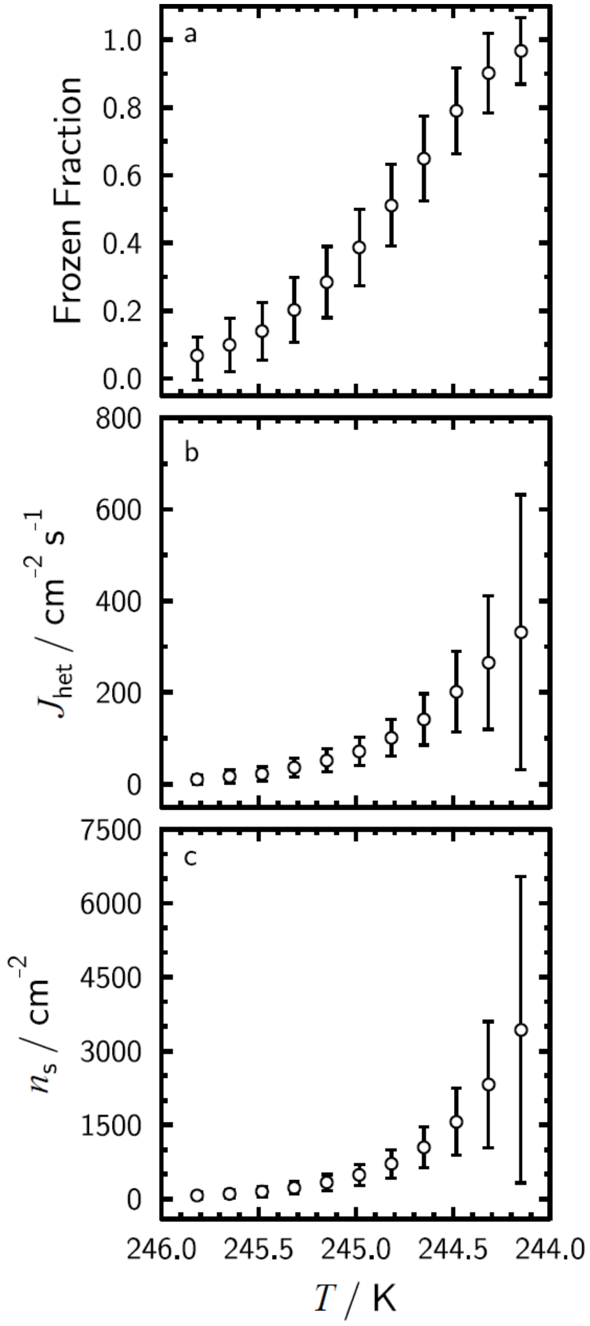

Figure S5| Uncertainty analysis following error propagation through quadrature. Example frozen fraction values derived from a cooling rate simulation using 100 droplets and identical surface area per droplet, A=0.001 cm^2, are shown in panel (a). The error on frozen fraction are exemplary and the error on A is assumed as 20%. Panel (b) and (c) show heterogeneous ice nucleation rate coefficients, J_{het}, and ice active surface site densities, n_s. Error in (b) and (c) are derived through quadrature.

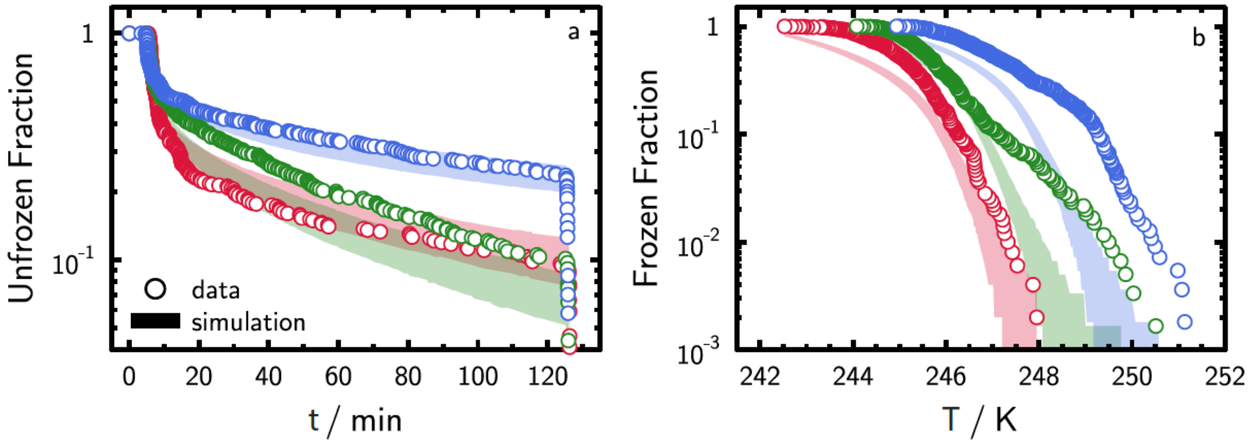

Figure S6| Experimentally derived (symbols) and simulated (shading) unfrozen fractions of isothermal freezing experiments (a) as a function of time, *t*, and frozen fractions of constant cooling rate freezing experiments (b) as a function of temperature, *T*, outlined in Table S1. Immersed surface area per droplet is unknown and uniformly distributed (Table S2) in model. Blue color represents ISO1 and CCR1, green color represents ISO2 and CCR2, and red color represents ISO3 and CCR3. Shadings represent the modeled 5th and 95th percentiles.

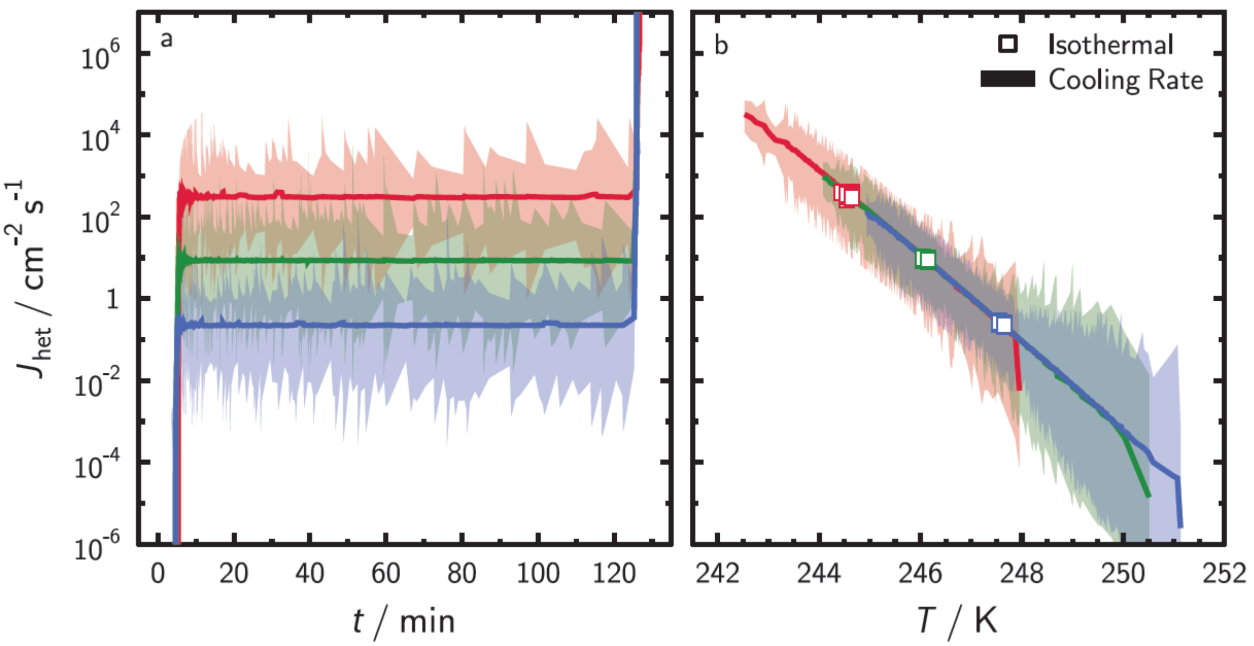

Figure S7| Derived heterogeneous ice nucleation rate coefficients, J_{het}, from simulation results for isothermal freezing experiments (a) as a function of time, *t*, and constant cooling rate freezing experiments (b) as a function of temperature, *T*, outlined in Table S1. Blue color represents ISO1 and CCR1, green color represents ISO2 and CCR2, and red color represents ISO3 and CCR3. Immersed surface area per droplet is uniformly distributed (Table S2). Values of J_{het} for the isothermal experiment in (a) are shown as squares in (b) for comparison. The shadings in (a) and (b) are the upper and lower applied fiducial limits of observed freezing events for each recorded *t* or *T* interval.

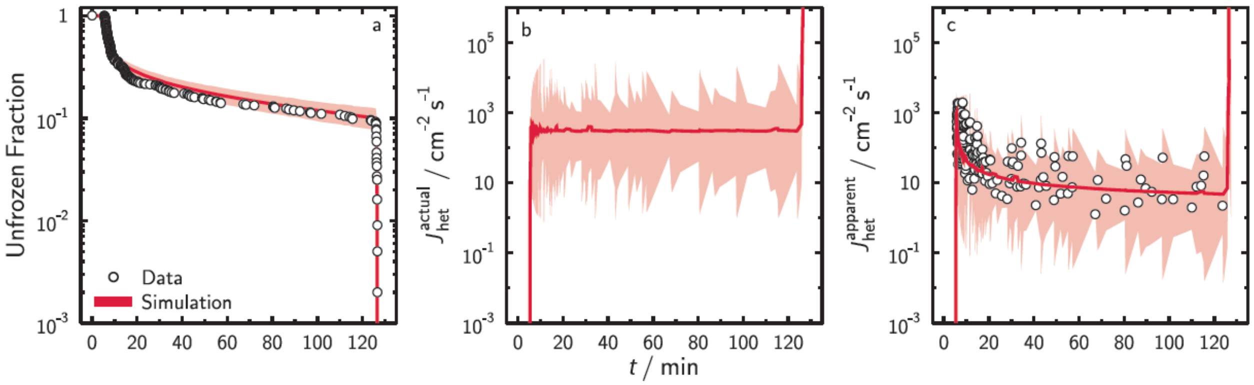

Figure S8| Evaluation of the assumption of identical or variable INP surface area (ISA) per droplet for isothermal freezing experiment, ISO3. (a) Experimentally derived and simulated unfrozen fraction of isothermal freezing experiment with 5th and 95th percentiles given by shading applying uniformly distributed ISA per droplet. Data in (a) is taken from Fig. 3a. (b) Calculated J_{het} using uniformly distributed ISA per droplet referred to as J_{het}^{actual}. The shading is the range of the upper and lower fiducial limits following Poission statistics at the 0.999 confidence level. Simulation results in (a) and (b) are taken from Figs. S6a and S7a, respectively. (c) Experimentally derived and recalculated J_{het} assuming identical ISA per droplet equal to measured mean BET values, termed $J_{het}^{apparent}$, is shown as open circles and shading, respectively. $J_{het}^{apparent}$ derived from the model was calculated from the modeled *UnF* in (a). The red solid lines are mean model values.

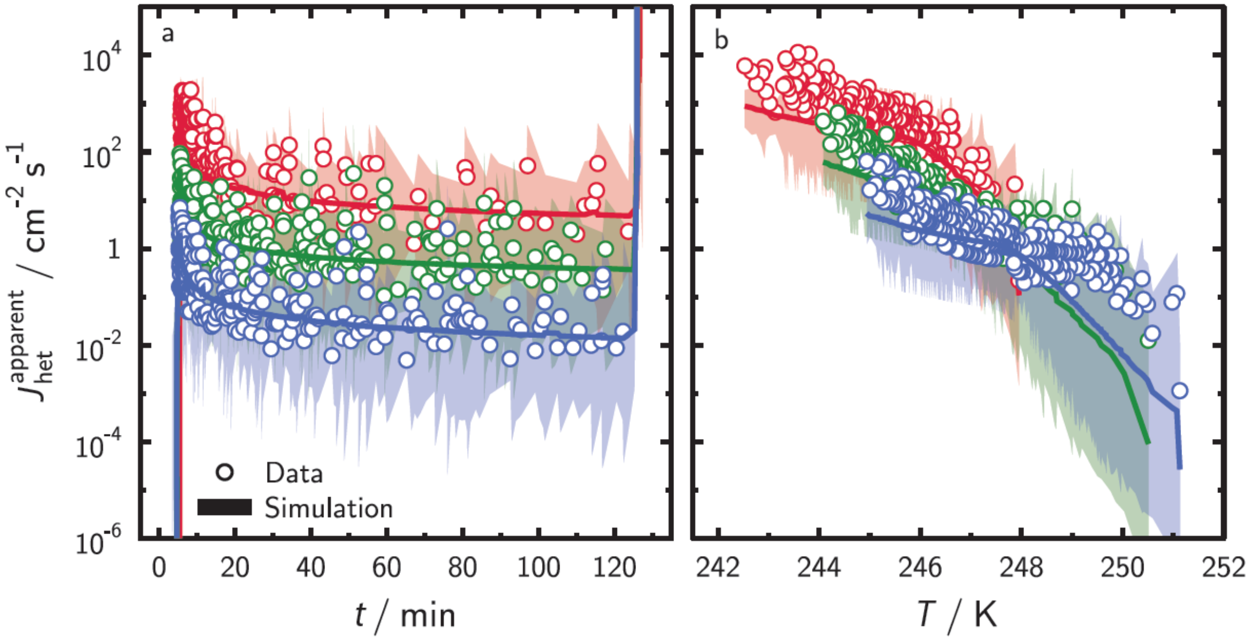

Figure S9| Evaluation of the assumption of identical or variable INP surface area (ISA) per droplet for all isothermal (a) and constant cooling rate (b) immersion freezing experiments as a function of time, *t*, or temperature, *T*, respectively, given in Table S1 for illite in water droplets. Blue color represents ISO1 and CCR1, green color represents ISO2 and CCR2, and red color represents ISO3 and CCR3. Symbols, lines and shading are the same as in Fig. S8c. $J_{het}^{apparent}$ derived from the model in (a) and (b) was calculated from the modeled *UnF* in Fig. S6(a) and the *FF* in Fig. S6(b) with uniformly distributed ISA however, using an incorrect assumption that droplets had identical ISA.

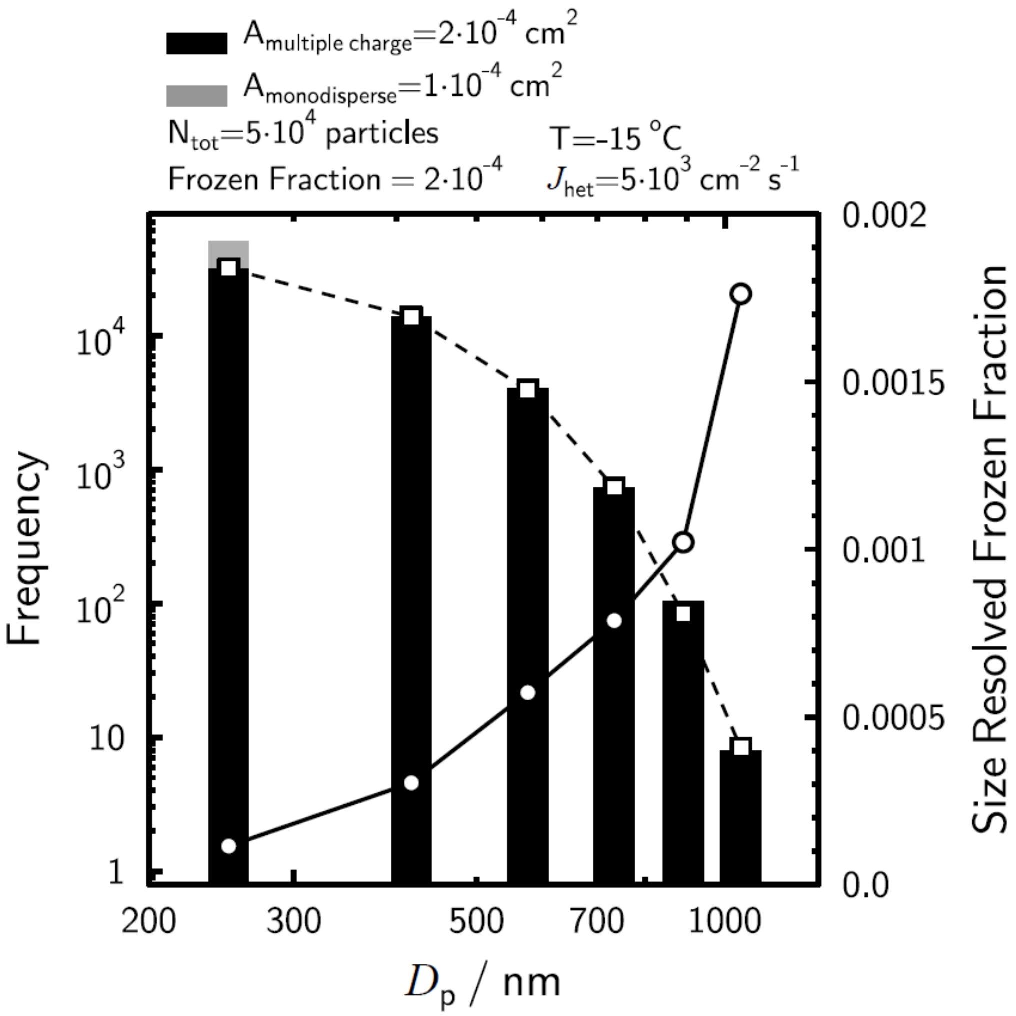

Figure S10| Randomly sampled aerosol particle size distribution (black bars) and size-discriminated contribution to observed frozen fraction (solid line and circles) due to the presence of multiple charged particles when selecting 250 nm size particles by an electrostatic particle size classifier based on the bipolar particle charge distribution[20]. A total of 5×10^4 particles were sampled. The expected frequency distribution is given by squares connected by dashed lines. The total particle surface area present is 1×10^{-4} and 2×10^{-4} cm^2 when assuming a monopolar (grey bar) and bipolar charge distribution, respectively. The size-discriminated frozen fraction was determined from the modeled frozen fraction of 2×10^{-4} with a heterogeneous ice nucleation rate coefficient, $J_{het} = 5\times10^3$ cm^{-2} s^{-1}. The analysis applies an ice nucleation activation time of 10 s.

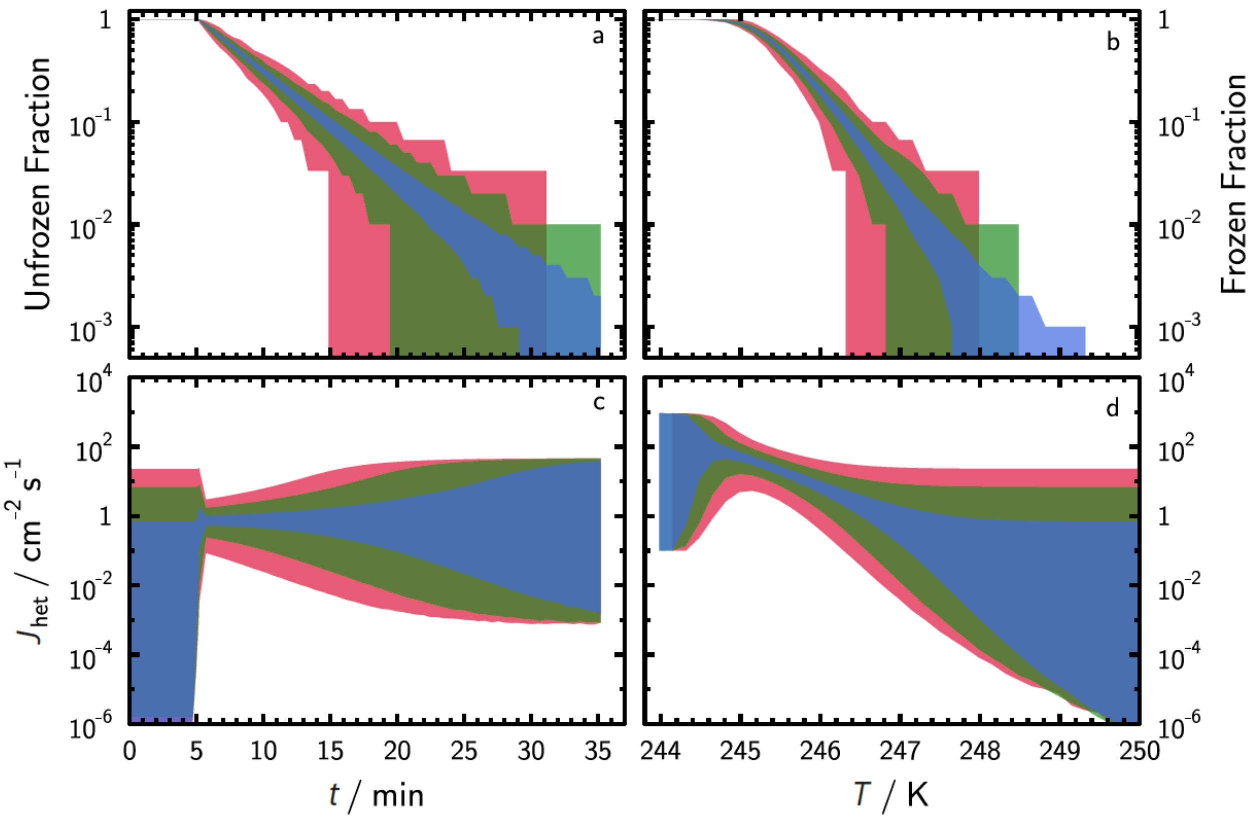

Figure S11| Sensitivity of uncertainties in unfrozen fraction, frozen fraction, and heterogeneous ice nucleation rate coefficients, J_{het}, using identical ISA applied in isothermal (a, c) and constant cooling rate (b, d) immersion freezing simulations using 30, 100, and 1000 observed freezing events as red, green and blue colors. Shading in unfrozen and frozen fraction curves in (a) and (b), respectively, are 5th and 95th percentiles. Shading in (c) and (d) are upper and lower fiducial limits of the derived J_{het} values.

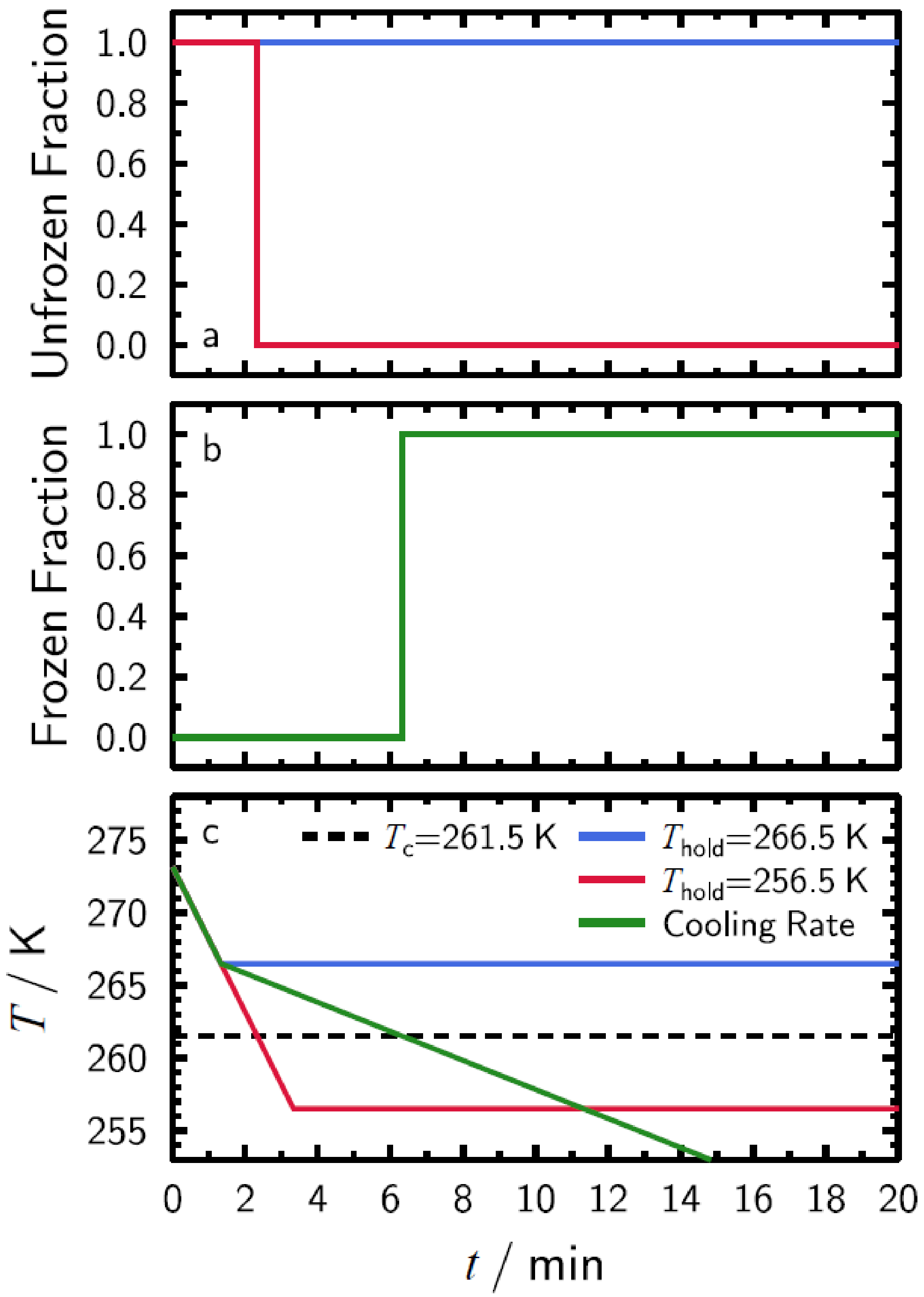

Figure S12| Predicted unfrozen and frozen fractions from isothermal (ISO) and constant cooling rate (CCR) immersion freezing experiments having particles with perfect ice active sites, i.e., all active sites form ice at the same characteristic temperature, T_c. (a) ISO freezing experiments showing unfrozen droplet fraction when temperature, T, is held at warmer (blue) or colder (red) T than T_c. (b) A CCR freezing experiment (green) during which T decreases below T_c. Panel (c) shows the experimental T profiles over time, t, for ISO freezing experiments (blue and red) shown in (a) and CCR freezing experiments (green) shown in (b). The temperature at ISO conditions, T_{hold}, is indicated along with T_c.

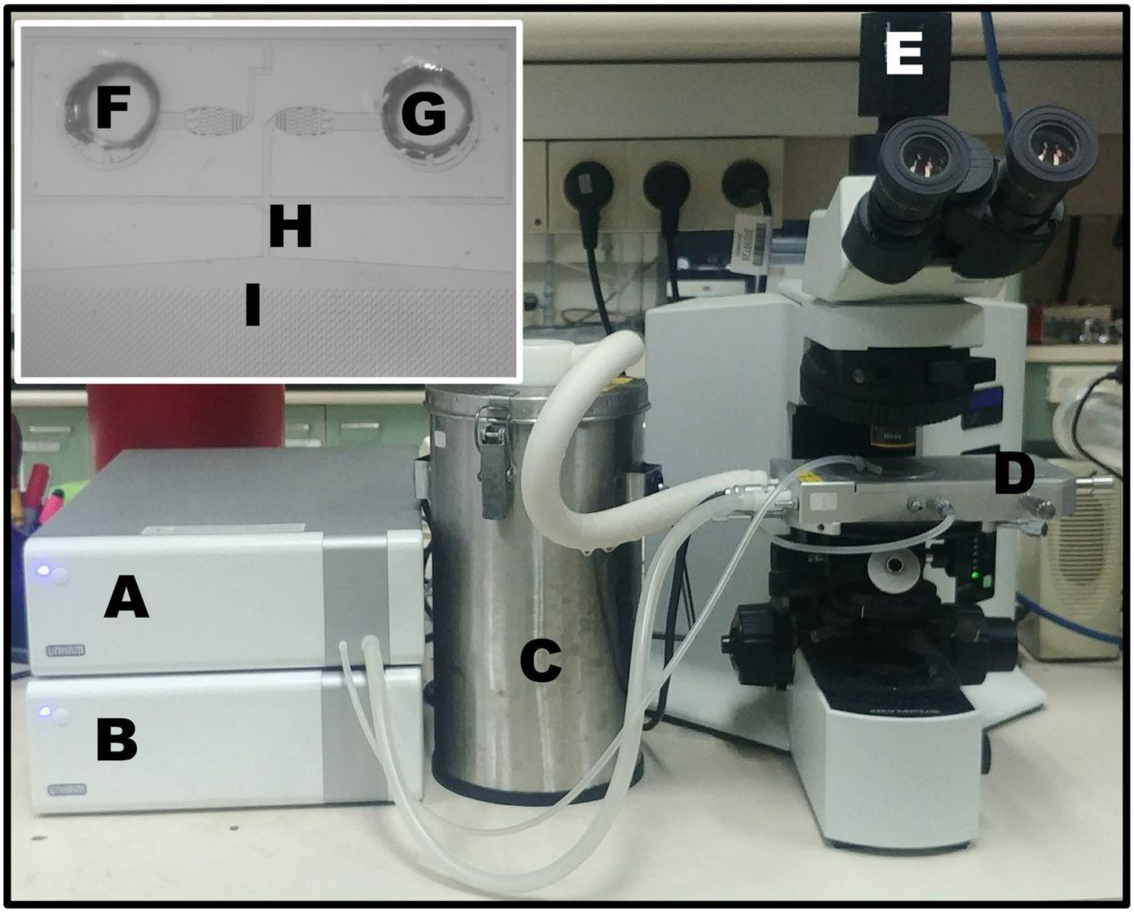

Figure S13| The WISDOM setup and the microfluidic chip (shown in upper left panel). A and B: pumps; C: liquid nitrogen tank; D: Linkam cooling stage; E: CCD camera; F and G: oil and dust/water inlets, respectively; H: drops generation point; I: channels with traps for the drops.

Supplementary Tables

Table S1 Details on the different ice nucleation experiments of this paper.

Experiment Name	Experiment Description	# of drops	Mean D (σ) / μm	T_{50} (σ) / K	T_{10} (σ) / K	T_{hold} / K	Mean SA / cm^2
CCR-DIW	DIW- Constant cooling	380	86.2 (4)	236.66 (<0.05)	237.15 (<0.05)	-	-
				0.60			
ISO-DIW	DIW- Isothermal	383	85.5 (5)	-	-	237.15	-
CCR1	Illite-NX, 1wt% Constant cooling	554	91.2 (3)	247.13 (0.38)	249.24 (0.26)	-	4.31×10^{-3}
ISO1	Illite-NX, 1wt% Isothermal	484	91.8 (3.8)	-	-	247.65	4.40×10^{-3}
CCR2	Illite-NX, 0.1wt% Constant cooling	601	91.0 (3.7)	245.06 (0.22)	246.23 (0.13)	-	4.29×10^{-4}
ISO2	Illite-NX, 0.1wt% Isothermal	508	90.1 (3.6)	-	-	246.15	4.16×10^{-4}
CCR3	Illite-NX, 0.005wt% Constant cooling	499	88.5 (4.7)	245.65 (0.22)	247.16 (0.48)	-	1.97×10^{-5}
ISO3	Illite-NX, 0.005wt% Isothermal	437	91.0 (3.7)	-	-	244.65	2.14×10^{-5}

Table S2 Parameters used in immersion freezing simulations. Following a water activity-based ice nucleation theory[8], $\log_{10}J_{het}=m\Delta a_w+c$. Immersed ice nucleating particle surface area (ISA) distributions were centered around the mean surface area (Table S1). For a lognormal distribution, the corresponding normal distribution has a standard deviation, σ. A range of ISA values at ±1σ is given. For a uniform distribution, the ISA was varied between ±σ orders of magnitude. The minimum and maximum ISA are given.

		CCR1 and ISO1	CCR2 and ISO2	CCR3 and ISO3
Lognormal Distribution Parameters	m	137.4	137.4	137.4
	c	-31.1	-31.1	-31.1
	σ	3.7	2.3	2.8
	±1σ range / cm^2	1×10^{-4} – 2×10^{-1}	4×10^{-5} – 4×10^{-3}	1×10^{-6} – 4×10^{-4}
Uniform Distribution Parameters	m	140	140	140
	c	-31.5	-31.5	-31.5
	σ	2	1.7	2.3
	A_{min} – A_{max} / cm^2	4×10^{-5} – 4×10^{-1}	8×10^{-6} – 2×10^{-2}	1×10^{-7} – 4×10^{-3}

Supplementary References

1 Knopf, D. A., Alpert, P. A. & Wang, B. The Role of Organic Aerosol in Atmospheric Ice Nucleation: A Review. *ACS Earth Space Chem.* **2**, 168–202, doi:10.1021/acsearthspacechem.7b00120 (2018).

2 Pruppacher, H. R. & Klett, J. D. *Microphysics of Clouds and Precipitation*. (Kluwer Academic Publishers, Dordrecht, 1997).

3 Koop, T., Luo, B. P., Biermann, U. M., Crutzen, P. J. & Peter, T. Freezing of $HNO_3/H_2SO_4/H_2O$ solutions at stratospheric temperatures: Nucleation statistics and experiments. *J. Phys. Chem. A* **101**, 1117-1133 (1997).

4 Seinfeld, J. H. & Pandis, S. N. *Atmospheric Chemistry and Physics: From Air Pollution to Climate Change*. 2nd edn, 1232 (Wiley-Interscience, Hoboken, 2006).

5 Knopf, D. A., Koop, T., Luo, B. P., Weers, U. G. & Peter, T. Homogeneous nucleation of NAD and NAT in liquid stratospheric aerosols: insufficient to explain denitrification. *Atmos. Chem. Phys.* **2**, 207-214 (2002).

6 Zobrist, B., Koop, T., Luo, B. P., Marcolli, C. & Peter, T. Heterogeneous ice nucleation rate coefficient of water droplets coated by a nonadecanol monolayer. *J. Phys. Chem. C* **111**, 2149-2155, doi:Doi 10.1021/Jp066080w (2007).

7 Knopf, D. A. & Forrester, S. M. Freezing of Water and Aqueous NaCl Droplets Coated by Organic Monolayers as a Function of Surfactant Properties and Water Activity. *J. Phys. Chem. A* **115**, 5579-5591 (2011).

8 Knopf, D. A. & Alpert, P. A. A water activity based model of heterogeneous ice nucleation kinetics for freezing of water and aqueous solution droplets. *Faraday Discuss.* **165**, 513-534, doi:10.1039/C3FD00035D (2013).

9 Rigg, Y. J., Alpert, P. A. & Knopf, D. A. Immersion freezing of water and aqueous ammonium sulfate droplets initiated by humic-like substances as a function of water activity. *Atmos. Chem. Phys.* **13**, 6603-6622, doi:10.5194/acp-13-6603-2013 (2013).

10 Alpert, P. A. & Knopf, D. A. Analysis of isothermal and cooling-rate-dependent immersion freezing by a unifying stochastic ice nucleation model. *Atmos. Chem. Phys.* **16**, 2083-2107, doi:10.5194/acp-16-2083-2016 (2016).

11 Alpert, P. A., Aller, J. Y. & Knopf, D. A. Ice nucleation from aqueous NaCl droplets with and without marine diatoms. *Atmos. Chem. Phys.* **11**, 5539–5555, doi:10.5194/acp-11-5539-2011 (2011).

12 Reicher, N., Segev, L. & Rudich, Y. The WeIzmann Supercooled Droplets Observation on a Microarray (WISDOM) and application for ambient dust. *Atmos. Meas. Tech.* **11**, 233-248, doi:10.5194/amt-11-233-2018 (2018).

13 Riechers, B., Wittbracht, F., Hutten, A. & Koop, T. The homogeneous ice nucleation rate of water droplets produced in a microfluidic device and the role of temperature uncertainty. *Phys. Chem. Chem. Phys.* **15**, 5873-5887, doi:10.1039/c3cp42437e (2013).

14 Murray, B. J. *et al.* Kinetics of the homogeneous freezing of water. *Phys. Chem. Chem. Phys.* **12**, 10380–10387, doi:10.1039/c003297b (2010).

15 Knopf, D. A. & Rigg, Y. J. Homogeneous Ice Nucleation From Aqueous Inorganic/Organic Particles Representative of Biomass Burning: Water Activity, Freezing Temperatures, Nucleation Rates. *J. Phys. Chem. A* **115**, 762-773, doi:10.1021/jp109171g (2011).

16 Ickes, L., Welti, A., Hoose, C. & Lohmann, U. Classical nucleation theory of homogeneous freezing of water: thermodynamic and kinetic parameters. *Phys. Chem. Chem. Phys.* **17**, 5514-5537, doi:10.1039/c4cp04184d (2015).

17 Pruppacher, H. R. A New Look At Homogeneous Ice Nucleation in Supercooled Water Drops. *J. Atmos. Sci.* **52**, 1924-1933 (1995).

18 Koop, T. & Murray, B. J. A physically constrained classical description of the homogeneous nucleation of ice in water. *J. Chem. Phys.* **145**, 211915, doi:10.1063/1.4962355 (2016).

19 Herbert, R. J., Murray, B. J., Dobbie, S. J. & Koop, T. Sensitivity of liquid clouds to homogenous freezing parameterizations. *Geophys. Res. Lett.* **42**, 1599-1605, doi:10.1002/2014gl062729 (2015).
20 Tigges, L., Jain, A. & Schmid, H. J. On the bipolar charge distribution used for mobility particle sizing: Theoretical considerations. *J. Aerosol. Sci.* **88**, 119-134, doi:10.1016/j.jacrosci.2015.05.010 (2015).
